# In Vitro Enzymatic and Computational Assessments of Pyrazole–Isatin and Pyrazole–Indole Conjugates as Anti-Diabetic, Anti-Arthritic, and Anti-Inflammatory Agents

**DOI:** 10.3390/pharmaceutics17030293

**Published:** 2025-02-23

**Authors:** Ahmed M. Naglah, Abdulrahman A. Almehizia, Mohammed Ghazwani, Asma S. Al-Wasidi, Abdelrahman A. Naglah, Wael M. Aboulthana, Ashraf S. Hassan

**Affiliations:** 1 Drug Exploration & Development Chair (DEDC), Department of Pharmaceutical Chemistry, College of Pharmacy, King Saud University, Riyadh 11451, Saudi Arabia; mehizia@ksu.edu.sa; 2 Department of Pharmaceutics, College of Pharmacy, King Khalid University, P.O. Box 1882, Abha 61441, Saudi Arabia; myghazwani@kku.edu.sa; 3 Department of Chemistry, College of Science, Princess Nourah Bint Abdulrahman University, Riyadh 11671, Saudi Arabia; asalwasidi@pnu.edu.sa; 4 Faculty of Medicine, Zagazig University, Zagazig 44519, Egypt; boodynaglah@gmail.com; 5 Biochemistry Department, Biotechnology Research Institute, National Research Centre, Dokki, Cairo 12622, Egypt; wm.kamel@nrc.sci.eg; 6 Organometallic and Organometalloid Chemistry Department, National Research Centre, Dokki, Cairo 12622, Egypt

**Keywords:** molecular hybridization, anti-arthritic activity, cyclooxygenase-2 enzyme, toxicity class, drug-likeness model scores, polar surface area maps

## Abstract

**Background/Objectives**: Recently, the prevalence of diseases such as diabetes, arthritis, and inflammatory diseases, along with their complications, has become a significant health problem. This is in addition to the various biomedical applications of pyrazole, isatin, and indole derivatives. Accordingly, cooperation will continue between chemistry scientists, pharmaceutical scientists, and human doctors to produce hybrid compounds from pyrazole with isatin or indole possessing biological activities as anti-diabetic, anti-arthritic, and anti-inflammatory agents. **Methods**: The two series of pyrazole–isatin conjugates **12a**–**h** and pyrazole–indole conjugates **14a**–**d** were prepared from our previous works via the direct reaction of 5-amino-pyrazoles **10a**–**d** with *N*-alkyl isatin **11a**,**b**, and 1*H*-indole-3-carbaldehyde (**13**), respectively, using the previously reported procedure. The potential biological activities of **12a**–**h** and **14a**–**d** as anti-diabetic, anti-arthritic, and anti-inflammatory agents were assessed through estimated inhibition percentage (%) and the median inhibitory concentrations (IC_50_) using methods described in the literature. Further, the computational assessments of **12a**–**h** and **14a**–**d** such as toxic doses (the median lethal dose, LD_50_), toxicity classes, drug-likeness model scores (DLMS), molecular lipophilicity potential (MLP) maps, polar surface area (PSA) maps, and topological polar surface area (TPSA) values were predicted using available free websites. **Results**: The in vitro enzymatic assessment results showed that pyrazole–indole conjugate **14b** possesses powerful activities against (i) α-amylase (% = 65.74 ± 0.23, IC_50_ = 4.21 ± 0.03 µg/mL) and α-glucosidase (% = 55.49 ± 0.23, IC_50_ = 2.76 ± 0.01 µg/mL); (ii) the protein denaturation enzyme (% = 49.30 ± 0.17) and against the proteinase enzyme (% = 46.55 ± 0.17) with an IC_50_ value of 6.77 ± 0.01 µg/mL; (iii) the COX-1, COX-2, and 5-LOX enzymes with an IC_50_ of 5.44 ± 0.03, 5.37 ± 0.04, and 7.52 ± 0.04, respectively, which is almost close to the IC_50_ of the indomethacin and zileuton drugs. Also, the computational assessment results showed (i) the conjugate **14b** possesses lipophilic surface properties thus can cross cell membranes, and is effective for treatment; (ii) all the conjugates possess a TPSA value of more than 140 Å^2^ thus possess good intestinal absorption. **Conclusions**: The two series of pyrazole–isatin conjugates **12a**–**h** and pyrazole–indole conjugates **14a**–**d** were synthesized from our previous works. The results of these in vitro enzymatic and computational assessments concluded that the pyrazole–indole conjugate **14b** possesses powerful activities against various studied enzymes and possesses good computational results. In the future, our research team will present in vitro, in vivo biological, and computational assessments to hopefully obtain effectual agents such as anti-diabetic, anti-arthritic, and anti-inflammatory.

## 1. Introduction

Diabetes is a modern-day challenge caused by a malfunction in insulin secretion from the pancreas [1]. Insulin regulates glucose levels in the body [2]. Type 1 diabetes (T1D) is an autoimmune disease that usually affects children and young adults. It occurs when the immune system attacks the insulin-producing beta cells in the pancreas, leading to a lack of insulin production. This results in high blood sugar levels (hyperglycemia) as glucose cannot be properly transported into tissues. Type 2 diabetes (T2DM) is a common form of diabetes characterized by high blood sugar levels due to reduced insulin sensitivity and secretion, often influenced by lifestyle factors [3]. The protocols for α-amylase and α-glucosidase inhibitors are used in the treatment of diabetes. Acarbose acts as an inhibitor for α-amylase and α-glucosidase enzymes [4].

Oxidative stress (OS) is caused by free radicals, which can result in damage to cells and tissues. Complications related to oxidative stress include cancer, osteoarthritis, diabetes mellitus, and inflammatory diseases [5,6]. Rheumatoid arthritis (RA) is a type of inflammatory disease that results from a chronic condition [7]. The inhibition protocols of proteinase denaturation and proteinase are used in the treatment of arthritis [8]. Anti-inflammatory agents work by inhibiting COX-2, COX-1, and 5-LOX, making them effective strategies for treating inflammation [9].

Recently, molecular hybridization has become one of the important tools for drug discovery [10], involving the merging of two or more pharmacophores that possess biological activities into a single molecule [11]. For instance, Abdelazeem et al. [12] reported the synthesis of some sulfonamide–pyrazole hybrids as anti-diabetic and anti-Alzheimer agents. Hassan et al. [13] reported the synthesis of novel pyrazoline-benzofuran-pyrazole derivatives that exhibited powerful cytotoxicity. Shaldam et al. [14] reported the synthesis of isatin–sulfonamide hybrids as anticancer agents through the inhibitory effect against the VEGFR-2 enzyme.

**Figure 1:**
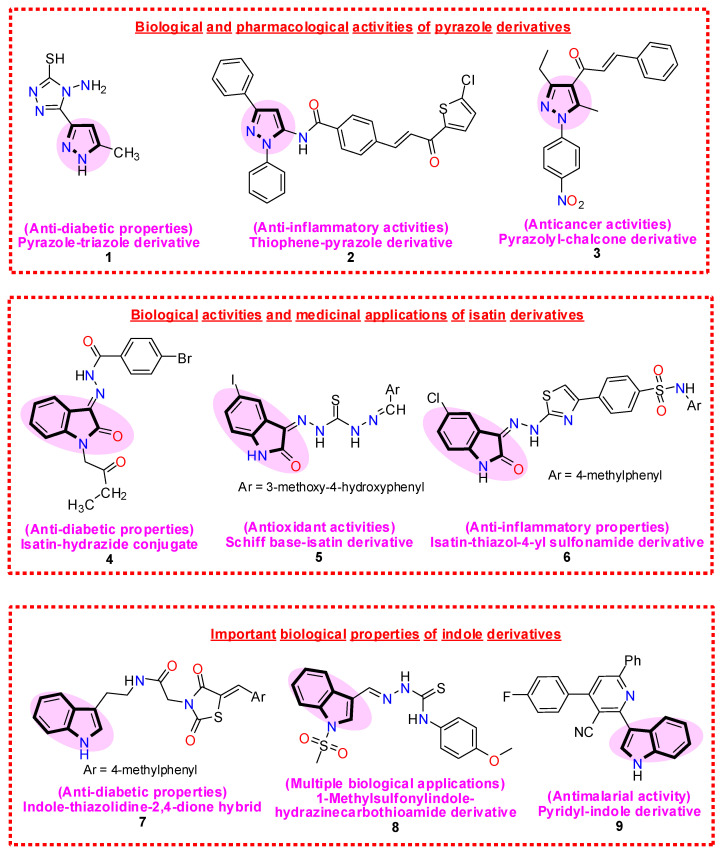
Examples of bioactive pyrazole **1**–**3**, isatin **4**–**6**, and indole **7**–**9** compounds.

In the last five years, pyrazole derivatives have demonstrated significant biological and pharmacological activities [15,16,17]. For example, pyrazole-triazole derivative **1** (IC_50_ = 95.85 ± 0.92 µM) possesses anti-diabetic properties against the α-glucosidase enzyme [18]. Thiophene-pyrazole derivative **2** demonstrated powerful anti-inflammatory activities against cyclooxygenase-1 (COX-1) enzyme with an IC_50_ of 10.21 µM and against cyclooxygenase-2 (COX-2) enzyme with an IC_50_ of 1.76 µM [19]. The pyrazolyl-chalcone derivative 3 demonstrated powerful anticancer activities against pancreatic cancer (PaCa-2) cells with an IC_50_ of 13.0 μg/mL and it also induces cell cycle arrest at the S and G2/M phases in the treated PaCa-2 cells and causes DNA damage [20] (Figure 1).

On the other hand, isatin derivatives possess biological activities and medicinal applications [20,21,22]. Isatin-hydrazide conjugate **4** possesses significant anti-diabetic properties against α-amylase enzyme with an IC_50_ of 19.6 µg/mL and also against α-glucosidase with an IC_50_ of 14.8 µg/mL [23]. Schiff base-isatin derivative **5** showed antioxidant activities with an IC_50_ of 9.76 ± 0.03 µM [24]. The isatin-thiazol-4-yl sulfonamide derivative **6** possesses significant anti-inflammatory properties with edema inhibition (EI = 38.50%). Additionally, derivative 6 showed potent inhibition with selectivity against the COX-2 enzyme, with an IC_50_ of 8.26 µM and an SI of 2.65. Furthermore, it demonstrated a good fit in the COX-2 binding site with a docking energy score of −11.45 kcal/mol [25] (Figure 1).

Moreover, indoles are privileged scaffolds in numerous derivatives with important biological properties [26,27,28,29,30]. The indole–thiazolidine-2,4-dione hybrid **7** displayed powerful α-glucosidase inhibitory activity with an IC_50_ of 2.35 ± 0.11 μM. It also suppressed glucose levels in diabetic mice through in vivo anti-diabetic investigations [31]. The 1-methylsulfonylindole-hydrazinecarbothioamide derivative **8** possesses multiple biological applications. It demonstrates selective antibacterial activity against Gram-negative bacteria, antioxidant activity, and anti-inflammatory activity by reducing TNF-α levels, and high power towards COX-2 enzyme inhibition [32]. The pyridyl-indole derivative **9** exhibited antimalarial activity [33] (Figure 1).

**Figure 2:**
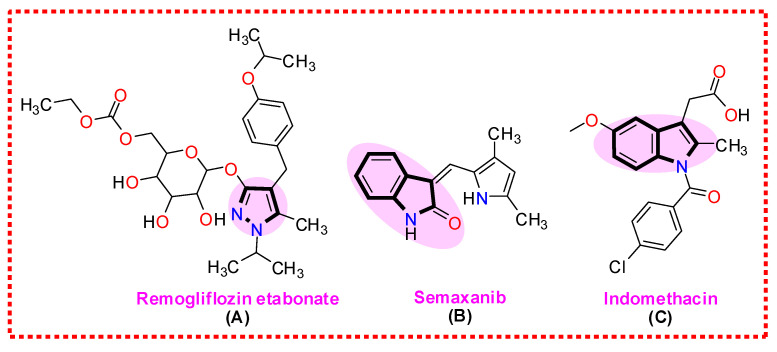
Example of pyrazole-based drug (**A**), isatine-based drug (**B**), and indole-based drug (**C**).

There are various pyrazole, isatin, or indole-based drugs available on the market, highlighting their significance in medicine and pharmaceutical chemistry [34,35,36,37]. Remogliflozin etabonate (**A**) is an example of a pyrazole-based drug. Remogliflozin etabonate possesses anti-diabetic properties that can be used for the treatment of Type 2 diabetes [38] and is a potent and selective inhibitor of the sodium-glucose co-transporter 2 (SGLT2) [39]. Semaxanib (**B**) is an example of an isatin-based drug used for the treatment of colorectal cancer and lung cancer that acts as a tyrosine kinase inhibitor [40]. Indomethacin (**C**) is an example of an indole-based drug. Indomethacin is a powerful nonsteroidal anti-inflammatory drug (NSAID) that possesses antipyretic, anti-inflammatory, and analgesic properties to treat various conditions [41] (Figure 2).

According to information on

(i) Diabetes mellitus and inflammatory diseases;

(ii) The various biological activities of pyrazole, isatin, and indole derivatives, particularly their anti-diabetic and anti-inflammatory properties;

(iii) Based on our previous studies focusing on the evaluation of compounds against diseases [42,43,44,45,46,47,48,49].

Based on the three points above, we have encouraged in this study for:

(1) Synthesizing two series of pyrazole–isatin conjugates **12a**–**h** and pyrazole–indole conjugates **14a**–**d** from our previous works.

(2) Evaluating their biological activities such as anti-diabetic, anti-arthritic, and anti-inflammatory activities.

(3) Performing the computational assessments including toxic doses (median lethal dose, LD_50_), toxicity classes, drug-likeness model scores (DLMS), molecular lipophilicity potential maps, polar surface area (PSA) maps, and topological polar surface area (TPSA) values.

## 2. Materials and Methods

### 2.1. Chemistry

5-Amino-pyrazoles **10a**–**d** [50], *N*-alkyl isatin **11a**,**b** [51], and the target conjugates, pyrazole–isatin conjugates **12a**–**h** and pyrazole–indole conjugates **14a**–**d**, were prepared according to the reported procedures from our previous works [52].

The procedures for pyrazole–isatin conjugates **12a**–**h** and pyrazole–indole conjugates **14a**–**d** prepared were provided in the Supplementary File [52]. Also, the chemical structure of the target conjugates was confirmed through their spectral data, which are provided in the Supplementary File [52]. Additionally, the charts of **12a**–**h** and **14a**–**d** were provided in the Supplementary File [52].

### 2.2. In Vitro Enzymatic Assessments

The anti-diabetic [53,54], anti-arthritic [55,56], and anti-inflammatory [57,58] potential activities of pyrazole–isatin conjugates **12a**–**h** and pyrazole–indole conjugates **14a**–**d** were evaluated by determining the inhibition percentage (%), and the median inhibitory concentrations (IC_50_) following documented procedures in the literature.

The in vitro enzymatic assessments approaches were provided in the Supplementary File.

Statistical analysis (see details in the Supplementary File).

### 2.3. Computational Assessments

Computational toxicity estimations of pyrazole–isatin conjugates **12a**–**h** and pyrazole–indole conjugates **14a**–**d** were predicted using the ProTox-3.0 website accessed on 27 October 2024 [59].

The drug-likeness model scores (DLMS) were predicted using the Molsoft web (https://www.molsoft.com/mprop/) accessed on 27 October 2024 [60].

The Molecular lipophilicity potential (MLP) [61] and the polar surface area (PSA) [62] maps were calculated using the Molinspiration Galaxy 3D Structure Generator v2023.08 available on the Molinspiration website: (https://www.molinspiration.com/cgi/galaxy) accessed on 21 October 2024.

Also, topological polar surface area (TPSA) values were predicted by using the Molinspiration website (https://www.molinspiration.com) accessed on 21 October 2024 [63].

## 3. Results and Discussion

### 3.1. Chemistry

**Scheme 1:**
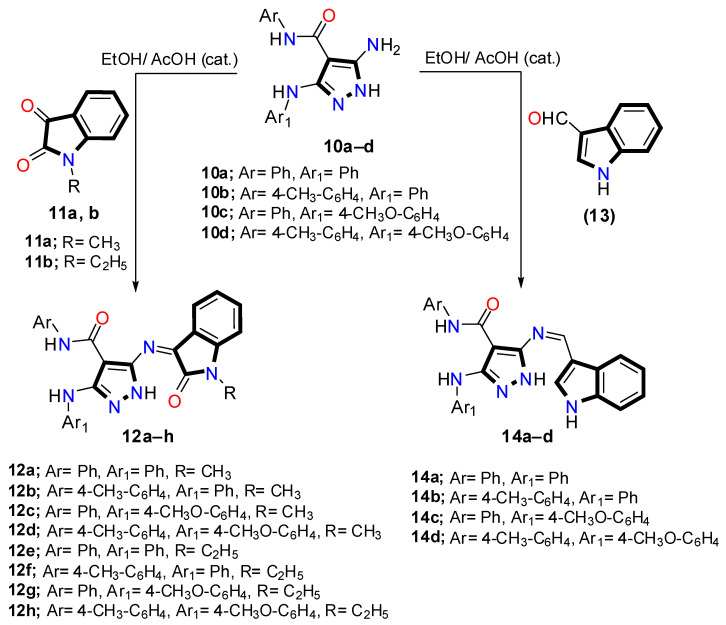
Synthesis of pyrazole–isatin **12a**–**h** and pyrazole–indole **14a**–**d.**

In this section, we describe the synthesis of two series from our previous works [52]: pyrazole–isatin conjugates **12a**–**h** and pyrazole–indole conjugates **14a**–**d** through direct reactions. The first series, pyrazole–isatin conjugates **12a**–**h**, was prepared by reacting 5-amino-pyrazoles **10a**–**d** with *N*-alkyl isatin **11a**,**b**. The second series, pyrazole–indole conjugates **14a**–**d**, was prepared by reacting 5-amino-pyrazoles **10a**–**d** with 1*H*-indole-3-carbaldehyde (**13**) [52] (Scheme 1).

All **12a**–**h** and **14a**–**d** compounds are known and reported from our previous works [52]. The hydrogen and carbon structures of the two series, **12a**–**h** and **14a**–**d**, were confirmed through spectral analyses [52]. The characterized spectral data and charts of the two series were provided in the Supplementary File [52].

### 3.2. In Vitro Enzymatic Assessments

#### 3.2.1. In Vitro Anti-Diabetic Assessment

In vitro anti-diabetic assessment of pyrazole–isatin **12a**–**h** and pyrazole–indole **14a**–**d** against α-amylase and α-glucosidase enzymes was assessed by using the reported methods in the literature [53,54]. The results of in vitro anti-diabetic assessment of pyrazole–isatin **12a**–**h** and pyrazole–indole **14a**–**d** are listed in Table 1.

**Figure 3:**
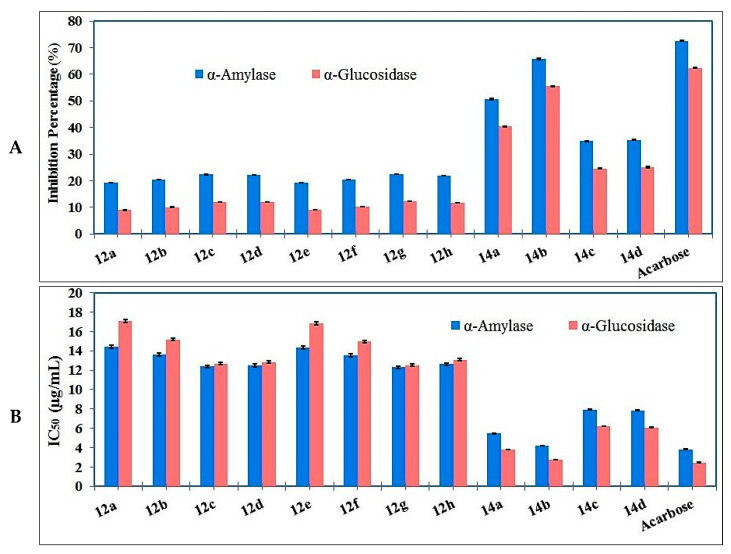
(**A**) The inhibition percentage (%) and (**B**) the median inhibitory concentrations (IC_50_) of the anti-diabetic activities of pyrazole–isatin **12a**–**h**, pyrazole–indole **14a**–**d,** and standard anti-diabetic agent (acarbose).

Figure 3A plots the inhibition percentage (%) and Figure 3B plots the median inhibitory concentrations (IC_50_) of the anti-diabetic activities of pyrazole–isatin **12a**–**h**, pyrazole–indole **14a**–**d**, and the standard anti-diabetic agent (acarbose).

In the case of the α-amylase enzyme, the inhibition percentage (%) of the pyrazole–isatin series **12a**–**h** ranged from 19.19 ± 0.03 to 22.47 ± 0.03 compared to acarbose (72.58 ± 0.16%) as the standard drug. Additionally, the median inhibitory concentrations (IC_50_) of the pyrazole–isatin series **12a**–**h** (from IC_50_ = 12.32 ± 0.13 to IC_50_ = 14.42 ± 0.16 µg/mL) were higher than the median inhibitory concentration of acarbose (IC_50_ = 3.81 ± 0.04 µg/mL). This indicates that this series is less active than acarbose against the α-amylase enzyme. Also, the pyrazole–indole series **14a**–**d** exhibits inhibition percentages ranging from 34.84 ± 0.23 to 65.74 ± 0.23. Additionally, it has median inhibitory concentrations (IC_50_) close to acarbose (IC_50_ = 3.81 ± 0.04 µg/mL). This signifies that this series is active against the α-amylase enzyme and pyrazole–indole **14b** is the most active among the two tested compounds, pyrazole–isatin **12a**–**h** and pyrazole–indole **14a**–**d**.

In the case of the α-glucosidase enzyme, the pyrazole–indole **14b** (55.49 ± 0.23%) showed a high inhibition percentage among the two tested series, pyrazole–isatin **12a**–**h** and pyrazole–indole **14a**–**d**, compared to acarbose (62.33 ± 0.16%) as the standard drug. Also, pyrazole–indole **14b** possesses IC_50_ of 2.76 ± 0.01 µg/mL which is close to acarbose (IC_50_ = 2.45 ± 0.02 µg/mL)

Finally, we can deduce that derivative **14b** possesses the most anti-diabetic activity among the two series tested against α-amylase and α-glucosidase enzymes.

The conjugate **14b** possesses two scaffolds, 4-methylphenyl and phenyl, which have a positive effect on carbohydrate-metabolizing enzymes. This result is consistent with previous findings by Nkoana et al. [64] and Sharma et al. [65] regarding the ability of these two scaffolds, 4-methyl phenyl and phenyl, to inhibit α-amylase and α-glucosidase enzymes.

#### 3.2.2. In Vitro Anti-Arthritic Assessment

In vitro anti-arthritic assessment of pyrazole–isatin **12a**–**h** and pyrazole–indole **14a**–**d** against protein denaturation and proteinase enzymes was estimated by using the methods reported in the literature [55,56]. The results of the in vitro anti-arthritic assessment of pyrazole–isatin **12a**–**h** and pyrazole–indole **14a**–**d** are listed in Table 2.

It was found that the pyrazole–indole **14b** (49.30 ± 0.17%) possesses a high inhibition percentage effect on protein denaturation compared to the proficiency of diclofenac sodium (48.75 ± 0.04%). Also, the same product, **14b**, exhibits a high inhibition percentage of 46.55 ± 0.17% and an IC_50_ value of 6.77 ± 0.01 µg/mL against the proteinase enzyme, which is more potent than diclofenac sodium (46.00 ± 0.04% and IC_50_ = 6.85 ± 0.022 µg/mL).

This signifies that this derivative **14b** possesses the most anti-arthritic activity against the two enzymes, protein denaturation and proteinase.

#### 3.2.3. In Vitro Anti-Inflammatory Assessment

The in vitro anti-inflammatory assessment of pyrazole–isatin **12a**–**h** and pyrazole–indole **14a**–**d** against cyclooxygenase-1 (COX-1), cyclooxygenase-2 (COX-2), and 5-lipoxygenase (5-LOX) enzymes was estimated by using the mentioned methods in the literature [57,58]. The results of the in vitro anti-inflammatory assessment of pyrazole–isatin **12a**–**h,** pyrazole–indole **14a**–**d,** and standard anti-inflammatory agents against COX-1, COX-2, and 5-LOX enzymes are recorded in Table 3.

**Figure 4:**
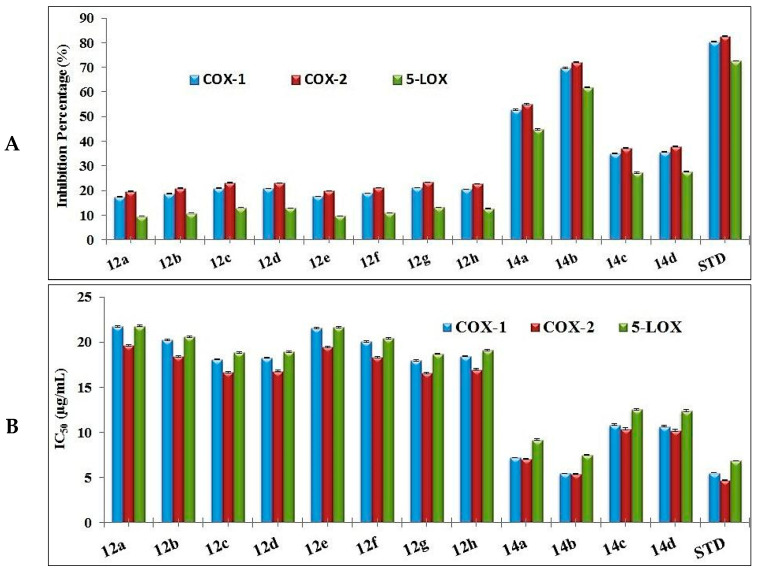
(**A**) The inhibition percentage (%) and (**B**) the median inhibitory concentrations (IC_50_) of anti-inflammatory assessments of pyrazole–isatin **12a**–**h**, pyrazole–indole **14a**–**d**, and standard anti-inflammatory agents.

Figure 4A plots the inhibition percentage (%) and Figure 4B plots the median inhibitory concentrations (IC_50_) of the anti-inflammatory assessments of pyrazole–isatin **12a**–**h**, pyrazole–indole **14a**–**d**, and standard anti-inflammatory agents. Indomethacin is the standard anti-inflammatory agent against COX-1 and COX-2 enzymes while zileuton is against the 5-LOX enzyme.

The results from Table 3, Figure 4A,B revealed that the pyrazole–isatin series **12a**–**h** possesses a weak range of inhibition percentages from 17.62 ± 0.04 to 21.15 ± 0.04 against the COX-1 enzyme, from 19.72 ± 0.04 to 23.40 ± 0.04 against the COX-2 enzyme, and from 9.57 ± 0.04 to 13.25 ± 0.04 against the 5-LOX enzyme. These ranges were compared with indomethacin as the standard drug with inhibition percentages of 80.51 ± 0.07 against the COX-1 enzyme and 82.76 ± 0.07 against the COX-2 enzyme and with zileuton as the standard drug with an inhibition percentage of 72.61 ± 0.07 against the 5-LOX enzyme. Also, the median inhibitory concentrations (IC_50_) of the pyrazole–isatin series **12a**–**h** are higher than the IC_50_ of indomethacin and zileuton; this refers to their weak activities as anti-inflammatory agents.

On the other hand, the pyrazole–indole series **14a**–**d** possesses high inhibition percentages against the COX-1, COX-2, and 5-LOX enzymes with ranges from 35.07 ± 0.26 to 69.83 ± 0.26, 37.32 ± 0.26 to 72.08 ± 0.26, and from 27.17 ± 0.26 to 61.93 ± 0.26, respectively, compared with the inhibition percentages of indomethacin against COX-1 (% = 80.51 ± 0.07) and COX-2 (% = 82.76 ± 0.07), and with the inhibition percentage of zileuton against 5-LOX (% = 72.61 ± 0.07). Additionally, the median inhibitory concentrations (IC_50_) of the pyrazole–indole series **14a**–**d** are lower than the IC_50_ of the indomethacin and zileuton; this refers to their powerful activities as anti-inflammatory agents.

Interestingly, the pyrazole–indole derivative **14b** possesses powerful anti-inflammatory activity against the COX-1, COX-2, and 5-LOX enzymes with an IC_50_ of 5.44 ± 0.03, 5.37 ± 0.04, and 7.52 ± 0.04, respectively. The IC_50_ of the pyrazole–indole derivative **14b** is almost closed with the IC_50_ of indomethacin (IC_50_ = 5.50 ± 0.01 for COX-1 enzyme and IC_50_ = 4.68 ± 0.02 for COX-2 enzyme) and zileuton (IC_50_ = 6.83 ± 0.02 for 5-LOX enzyme). The presence of the two scaffolds, 4-methylphenyl and phenyl, in compound **14b** has a clear effect on the inhibition of COX-1, COX-2, and 5-LOX enzymes [66].

Statistical analysis of the biological assessments was performed, and the results were recorded in Table 4. It was observed that the anti-diabetic assessment (inhibition % of α-amylase and α-glucosidase), anti-arthritic assessment (inhibition % of protein denaturation and proteinase), and anti-inflammatory assessments (inhibition % of COX-1, COX-2, and 5-LOX) are positively correlated with each other at *p* ≤ 0.05. When the anti-diabetic assessment increases, the anti-arthritic and anti-inflammatory assessments also increase.

### 3.3. Computational Assessments

#### 3.3.1. Toxicity Prediction

Computational toxicity estimations, such as toxic doses (the median lethal dose, LD_50_) and toxicity classes of pyrazole–isatin **12a**–**h** and pyrazole–indole **14a**–**d**, were predicted using the ProTox-3.0 website (https://tox-new.charite.de/protox_II/index.php?site=home) accessed on 27 October 2024 [59]. The computational toxicity estimation results are recorded in Table 5. These results indicate that the two target series, pyrazole–isatin **12a**–**h** and pyrazole–indole **14a**–**d**, have a median lethal dose (LD_50_) value of 1000 mg kg^−1^. As a result, both series were classified as class IV (300 < LD_50_ ≤ 2000) which refers to that harmful if swallowed.

#### 3.3.2. The Drug-Likeness Model Scores (DLMS) Prediction

The drug-likeness model scores (DLMS) of pyrazole–isatin conjugates **12a**–**h** and pyrazole–indole conjugates **14a**–**d** were predicted using the Molsoft web (https://www.molsoft.com/mprop/) accessed on 27 October 2024 [60]. The results of the computational DLMS are listed in Table 5.

The rule for estimating the drug-likeness model score of the target compounds is based on positive and negative values [67] as follows:

(i) The compound is considered drug-like if the drug-likeness score has a positive value.

(ii) The compound is considered non-drug-like if the drug-likeness score has a negative value.

**Figure 5:**
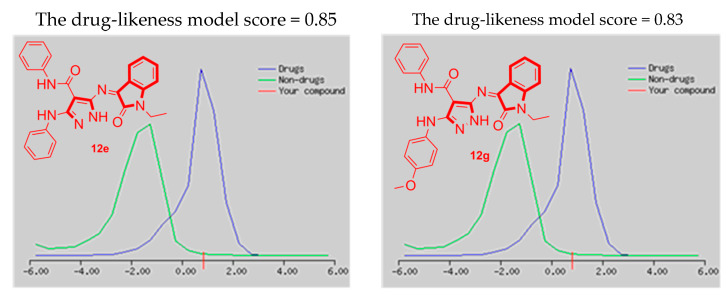
Displays the plotting of the drug-likeness model scores for **12e** and **12g**.

According to this rule, all pyrazole–isatin conjugates **12a**–**h** can be classified as drug-like compounds because these derivatives have positive drug-likeness scores ranging from 0.70 to 0.85. Therefore, the pyrazole–isatin conjugates **12a**–**h** show potential as drug candidates. Figure 5 represents the plotting of the drug-likeness model scores for the two pyrazole–isatin conjugates **12e** and **12g** which have the highest positive values of 0.85 and 0.83, respectively. Unfortunately, we found that the drug-likeness scores of the pyrazole–indole conjugates **14a**–**d** are negative, so this series is classified as non-drug-like candidates.

#### 3.3.3. Molecular Lipophilicity Potential (MLP) Maps

**Figure 6:**
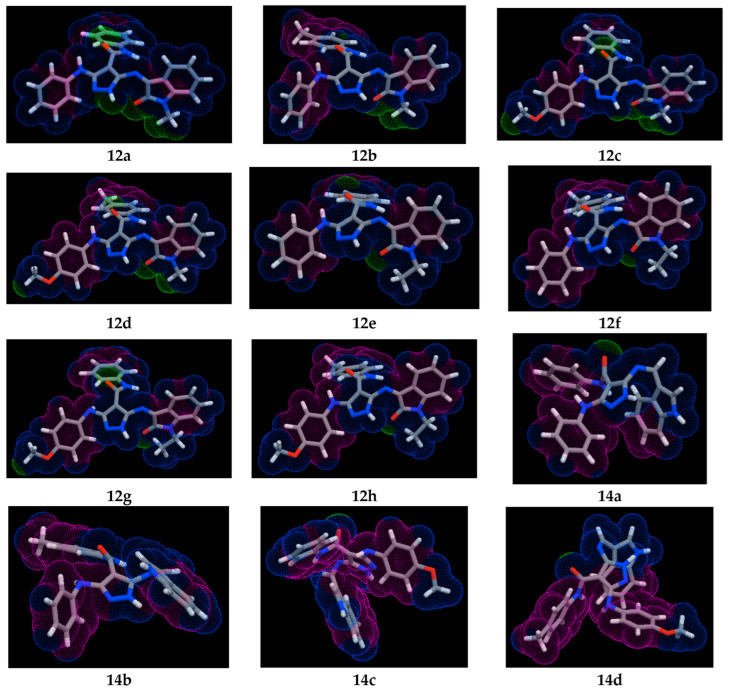
The MLP maps of pyrazole–isatin conjugates **12a**–**h** and pyrazole–indole conjugates **14a**–**d**.

The molecular lipophilicity potential (MLP) maps of pyrazole–isatin conjugates **12a**–**h** and pyrazole–indole conjugates **14a**–**d** were calculated using the Molinspiration Galaxy 3D Structure Generator v2023.08 available on the Molinspiration website: https://www.molinspiration.com/cgi/galaxy (accessed on 21 October 2024) [61]. Figure 6 displays the MLP maps of pyrazole–isatin conjugates **12a**–**h** and pyrazole–indole conjugates **14a**–**d**.

The molecular lipophilicity potential (MLP) maps on the molecular surfaces of pyrazole–isatin conjugates **12a**–**h** and pyrazole–indole conjugates **14a**–**d** indicates the lipophilic and hydrophilic parts. The different colors of the MLP maps refer to lipophilic and hydrophilic areas as follows [68]:

(i) The most lipophilic area is represented by a blue color.

(ii) The intermediate lipophilic area is represented by pink.

(iii) The most hydrophilic area is indicated by yellow.

(iv) The intermediate hydrophilic area is shown in green.

From Figure 6, which displays the molecular lipophilicity potential (MLP) maps of the conjugates **12a**–**h** and **14a**–**d**, we observed the following:

1. The three conjugates **12c**, **12d**, and **12g** exhibit similar lipophilic and hydrophilic properties, with the lipophilic regions covering most of the surface area except for three specific areas: the carbonyl of amide (-NH-CO-), CH_3_O-*p*-C_6_H_4_-NH-, and carbonyl of isatin (C=O).
2. The three conjugates **12a**, **12b**, and **12e** possess similar surface properties (lipophilic and hydrophilic). The intermediate hydrophilic area appears at the two positions: the carbonyl of amide (-NH-CO-) and the carbonyl of isatin (C=O).
3. The two conjugates **12f** and **12h** exhibit almost completely lipophilic properties, except for the carbonyl position of isatin (C=O), which shows intermediate hydrophilic properties.
4. The three conjugates **14a**, **14c**, and **14d** possess almost completely lipophilic surface properties, except for the carbonyl position of amide (-NH-CO-), which shows intermediate hydrophilic properties.
5. The pyrazole–indole conjugate **14b** possesses lipophilic surface properties.

The lipophilic surface area of the molecule increases its lipophilicity, enhancing its ability to penetrate cell membranes. Consequently, this molecule is more effective for treatment [69]. In this section, we found that the pyrazole–indole conjugate **14b** possesses completely lipophilic surface properties. Consequently, this conjugate **14b** has the ability to cross cell membranes and is effective for treatment.

#### 3.3.4. Polar Surface Area (PSA) Maps and Topological Polar Surface Area (TPSA)

The polar surface area (PSA) maps of pyrazole–isatin conjugates **14a**–**h** and pyrazole–indole conjugates **14a**–**d** were calculated using the Molinspiration Galaxy 3D Structure Generator v2023.08 available on the Molinspiration website: https://www.molinspiration.com/cgi/galaxy (accessed on 21 October 2024) [62]. Also, topological polar surface area (TPSA) values are related to polar surface area (PSA), which can predicted by using the Molinspiration website: https://www.molinspiration.com (accessed on 21 October 2024) [63].

**Figure 7:**
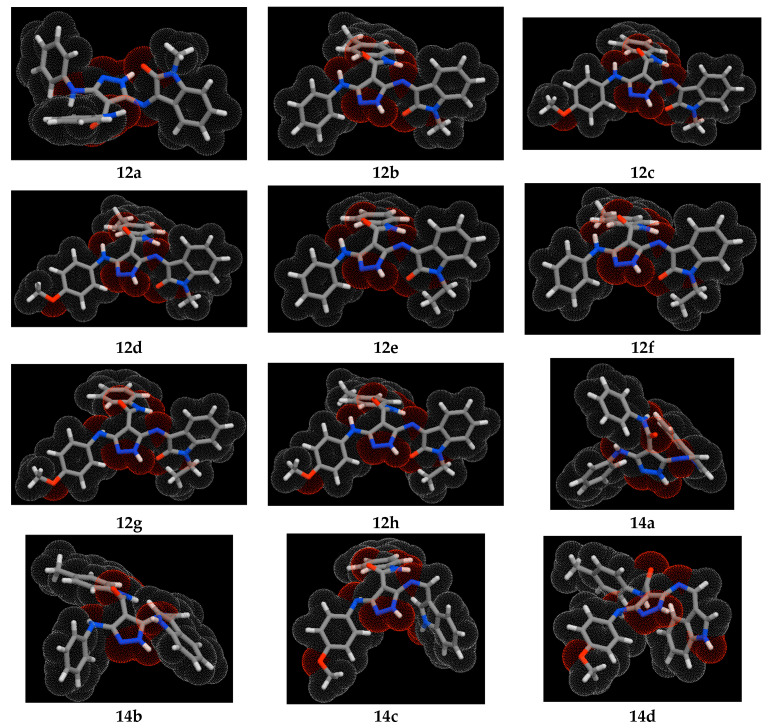
The PSA maps of pyrazole–isatin conjugates **12a**–**h** and pyrazole–indole conjugates **14a**–**d**.

Figure 7 displays the PSA maps of pyrazole–isatin conjugates **12a**–**h** and pyrazole–indole conjugates **14a**–**d**. While the topological polar surface area (TPSA) values of **12a**–**h** and **14a**–**d** are listed in Table 6.

The polar surface area (PSA) maps of pyrazole–isatin conjugates **12a**–**h** and pyrazole–indole conjugates **14a**–**d** indicate non-polar surface area and polar surface area. The two colors of the PSA maps refer to non-polar and polar areas as follows [61]:

(i) The non-polar area is represented by a gray color.

(ii) The polar area is represented by red.

From Figure 7, which displays the polar surface area (PSA) maps of the conjugates **12a**–**h** and **14a**–**d**, we observed that non-polar and polar areas are almost the same for the conjugates. The polar surface area is mainly found around heteroatoms (nitrogen and oxygen atoms) [70]. The polar surface area of the conjugates **12a**–**h** appears at the following positions: the amide (-NH-CO-), C_6_H_5_-NH- or CH_3_O-*p*-C_6_H_4_-NH-, =N-NH- of the pyrazole moiety, azomethine -N=C=, carbonyl of isatin (C=O), and N-CH_3_ or N-C_2_H_5_ of isatin. On the other hand, the polar surface area of the conjugates **14a**–**d** appears at the following positions: the amide (-NH-CO-), C_6_H_5_-NH-, CH_3_O-*p*-C_6_H_4_-NH-, =N-NH- of the pyrazole moiety, azomethine -N=CH-, and NH of indole.

Polar surface area (PSA) affects the molecule’s ability to permeate cells [71]. Thus, we predicted the topological polar surface area (TPSA) values through the Molinspiration website to determine this ability.

From Table 6, we observed that:

(i) The four conjugates (**12a, d, e,** and **f**) and the other four compounds (**12c**, **d**, **g**, and **h**) possess the same topological polar surface area (TPSA) values of 104.18 and 113.41 Å^2^, respectively.

(ii) The two conjugates, **14a** and **14b**, possess TPSA values of 97.69 Å^2^ while the other two conjugates, **14c** and **14d**, possess TPSA values of 107.20 Å^2^.

The TPSA influences intestinal absorption (IA) and penetration of the blood–brain barrier (BBB). The extent of IA and BBB penetration is determined by the TPSA values of the conjugates as follows [72]:

(i) The conjugates possess good intestinal absorption if the TPSA value is <140 Å^2^.

(ii) The conjugates possess good penetration of the blood–brain barrier if the TPSA value is <60 Å^2^.

According to the TPSA value rule, we can deduce that all the conjugates possess good intestinal absorption but do not possess sufficient blood–brain barrier penetration. This is consistent with previous discoveries on pyrazole conjugates which possess good human intestine absorption characteristics [73].

## 4. Conclusions

In summary, we synthesized two series of pyrazole–isatin conjugates **12a**–**h** and pyrazole–indole conjugates **14a**–**d** from our previous works. Both series were screened to evaluate their biological potentials in vitro for anti-diabetic, anti-arthritic, and anti-inflammatory applications.

The assessment results show that pyrazole–indole conjugate **14b** possesses powerful activities against the enzymes studied. Interestingly, pyrazole–indole **14b** showed a high inhibition percentage among the two tested series against α-amylase and α-glucosidase enzymes and possesses anti-α-glucosidase enzyme with an IC_50_ of 2.76 ± 0.01 µg/mL, which is close to acarbose (IC_50_ = 2.45 ± 0.02 µg/mL). The same derivative, **14b**, possesses an inhibition percentage of 49.30 ± 0.17 against protein denaturation compared to the proficiency of diclofenac sodium (48.75 ± 0.04%). It also exhibits inhibition (% = 46.55 ± 0.17) and an IC_50_ value of 6.77 ± 0.01 µg/mL against the proteinase enzyme, which is more potent than diclofenac sodium (46.00 ± 0.04% and IC_50_ = 6.85 ± 0.022 µg/mL). Finally, anti-inflammatory results indicate that the derivative **14b** possesses powerful anti-inflammatory activity against the COX-1, COX-2, and 5-LOX enzymes with an IC_50_ of 5.44 ± 0.03, 5.37 ± 0.04, and 7.52 ± 0.04, respectively, which is almost close to the IC_50_ of the indomethacin and zileuton drugs.

Also, computational toxicity estimations were predicted, and both series were classified as class IV (300 < LD_50_ ≤ 2000). The MLP map results indicate that the conjugate **14b** possesses lipophilic surface properties, can consequently cross cell membranes, and is effective for treatment. Additionally, the computational TPSA results indicate that all the conjugates possess a TPSA value of more than 140 Å^2^ thus possess good intestinal absorption.

Finally, after the enzymatic and computational assessment results of the two series, in the future, we can make modifications to their chemical structures and conduct more in vitro, in vivo biological and computational assessments to obtain an effective drug for treating inflammation diseases and diabetes.

## Figures and Tables

**Table 1:** The inhibition percentage (%) and IC_50_ values of in vitro anti-diabetic activity of pyrazole–isatin **12a**–**h**, pyrazole–indole **14a**–**d,** and acarbose against α-amylase and α-glucosidase enzymes.

Pyrazole Conjugates	α-Amylase		α-Glucosidase	
	Inhibition (%)	IC_50_ (µg/mL)	Inhibition (%)	IC_50_ (µg/mL)
**12a**	19.19 ± 0.03	14.42 ± 0.16	8.94 ± 0.03	17.10 ± 0.14
**12b**	20.33 ± 0.03	13.61 ± 0.15	10.08 ± 0.03	15.16 ± 0.12
**12c**	22.31 ± 0.03	12.40 ± 0.13	12.06 ± 0.03	12.68 ± 0.10
**12d**	22.16 ± 0.03	12.49 ± 0.14	11.91 ± 0.03	12.84 ± 0.10
**12e**	19.33 ± 0.03	14.32 ± 0.16	9.08 ± 0.03	16.85 ± 0.14
**12f**	20.48 ± 0.03	13.52 ± 0.15	10.23 ± 0.03	14.95 ± 0.12
**12g**	22.47 ± 0.03	12.32 ± 0.13	12.22 ± 0.03	12.52 ± 0.10
**12h**	21.94 ± 0.03	12.62 ± 0.14	11.69 ± 0.03	13.08 ± 0.10
**14a**	50.59 ± 0.23	5.47 ± 0.03	40.34 ± 0.23	3.79 ± 0.01
**14b**	65.74 ± 0.23	4.21 ± 0.03	55.49 ± 0.23	2.76 ± 0.01
**14c**	34.84 ± 0.23	7.94 ± 0.03	24.59 ± 0.23	6.22 ± 0.01
**14d**	35.34 ± 0.23	7.83 ± 0.04	25.09 ± 0.23	6.09 ± 0.01
**Acarbose**	72.58 ± 0.16	3.81 ± 0.04	62.33 ± 0.16	2.45 ± 0.02

Values were calculated from three replicates and expressed as the mean ± SE.

**Table 2:** The inhibition percentage (%) and IC_50_ values of in vitro anti-arthritic activity of pyrazole–isatin **12a**–**h**, pyrazole–indole **14a**–**d**, and diclofenac sodium against protein denaturation and proteinase enzymes.

Pyrazole Conjugates	Protein Denaturation	Proteinase	
	Inhibition (%)	Inhibition (%)	IC_50_ (µg/mL)
**12a**	14.40 ± 0.03	11.65 ± 0.03	23.20 ± 0.04
**12b**	15.25 ± 0.03	12.50 ± 0.03	21.90 ± 0.04
**12c**	16.73 ± 0.03	13.98 ± 0.03	19.96 ± 0.04
**12d**	16.62 ± 0.03	13.87± 0.03	20.10 ± 0.04
**12e**	14.50 ± 0.03	11.75 ± 0.03	23.04 ± 0.04
**12f**	15.36 ± 0.03	12.61 ± 0.03	21.75 ± 0.04
**12g**	16.85 ± 0.03	14.10 ± 0.03	19.82 ± 0.04
**12h**	16.45 ± 0.03	13.70 ± 0.03	20.30 ± 0.04
**14a**	37.94 ± 0.17	35.19 ± 0.17	8.80 ± 0.02
**14b**	49.30 ± 0.17	46.55 ± 0.17	6.77 ± 0.01
**14c**	26.13 ± 0.17	23.38 ± 0.17	12.78 ± 0.06
**14d**	26.50 ± 0.17	23.75 ± 0.17	12.60 ± 0.06
**Diclofenac Sodium**	48.75 ± 0.04	46.00 ± 0.04	6.85 ± 0.02

The results were calculated from three replicates and presented as the mean ± standard error (SE).

**Table 3:** The inhibition percentage (%) and IC_50_ values of in vitro anti-inflammatory activity of pyrazole–isatin **12a**–**h**, pyrazole–indole **14a**–**d**, and standard anti-inflammatory agents (indomethacin and zileuton) against COX-1, COX-2, and 5-LOX enzymes.

Pyrazole Conjugates	COX-1		COX-2		5-LOX	
	Inhibition (%)	IC_50_ (µg/mL)	Inhibition (%)	IC_50_ (µg/mL)	Inhibition (%)	IC_50_ (µg/mL)
**12a**	17.47 ± 0.04	21.74 ± 0.08	19.72 ± 0.04	19.64 ± 0.10	9.57 ± 0.04	21.79 ± 0.10
**12b**	18.75 ± 0.04	20.25 ± 0.07	21.00 ± 0.04	18.44 ± 0.09	10.85 ± 0.04	20.59 ± 0.09
**12c**	20.98 ± 0.04	18.10 ± 0.06	23.23 ± 0.04	16.68 ± 0.08	13.08 ± 0.04	18.83 ± 0.08
**12d**	20.80 ± 0.04	18.26 ± 0.06	23.05 ± 0.04	16.80 ± 0.08	12.90 ± 0.04	18.95 ± 0.08
**12e**	17.62 ± 0.04	21.55 ± 0.08	19.87 ± 0.04	19.49 ± 0.09	9.72 ± 0.04	21.64 ± 0.09
**12f**	18.91 ± 0.04	20.08 ± 0.07	21.16 ± 0.04	18.30 ± 0.09	11.01 ± 0.04	20.45 ± 0.09
**12g**	21.15 ± 0.04	17.95 ± 0.06	23.40 ± 0.04	16.55 ± 0.08	13.25 ± 0.04	18.70 ± 0.08
**12h**	20.56 ± 0.04	18.47 ± 0.06	22.81 ± 0.04	16.98 ± 0.08	12.66 ± 0.04	19.13 ± 0.08
**14a**	52.79 ± 0.26	7.19 ± 0.04	55.04 ± 0.26	7.04 ± 0.06	44.89 ± 0.26	9.19 ± 0.06
**14b**	69.83 ± 0.26	5.44 ± 0.03	72.08 ± 0.26	5.37 ± 0.04	61.93 ± 0.26	7.52 ± 0.04
**14c**	35.07 ± 0.26	10.83 ± 0.09	37.32 ± 0.26	10.38 ± 0.11	27.17 ± 0.26	12.53 ± 0.11
**14d**	35.63 ± 0.26	10.66 ± 0.09	37.88 ± 0.26	10.23 ± 0.11	27.73 ± 0.26	12.38 ± 0.11
**STD**	**Indomethacin**				**Zileuton**	
	80.51 ± 0.07	5.50 ± 0.01	82.76 ± 0.07	4.68 ± 0.02	72.61 ± 0.07	6.83 ± 0.02

The results were calculated from three replicates and presented as the mean ± standard error (SE).

**Table 4:** Significant correlations among measurements of the in vitro biological assessments of pyrazole–isatin **12a**–**h** and pyrazole–indole **14a**–**d**.

			Inhibition Percentage (%)						
			Anti-Diabetic		Anti-Arthritic		Anti-Inflammatory		
			α-Amylase	α-Glucosidase	Protein Denaturation	Proteinase	COX-1	COX-2	5-LOX
**Anti-diabetic assessment**	**Inhibition Percentage (%)**	**α-amylase**	-	0.000	0.000	0.000	0.000	0.000	0.000
		**α-Glucosidase**	0.000	-	0.000	0.000	0.000	0.000	0.000
**Anti-arthritic assessment**		**Protein Denaturation**	0.000	0.000	-	0.000	0.000	0.000	0.000
		**Proteinase**	0.000	0.000	0.000	-	0.000	0.000	0.000
**Anti-inflammatory assessment**		**COX-1**	0.000	0.000	0.000	0.000	-	0.000	0.000
		**COX-2**	0.000	0.000	0.000	0.000	0.000	-	0.000
		**5-LOX**	0.000	0.000	0.000	0.000	0.000	0.000	-

A significant correlation at *p* ≤ 0.05.

**Table 5:** The toxic doses, toxicity classes, and the drug-likeness model scores (DLMS) of pyrazole–isatin **12a**–**h** and pyrazole–indole **14a**–**d**.

Pyrazole Conjugates	Toxic Dose (LD_50_, mg kg^−1^)	Toxicity Class	Drug-Likeness Model Scores (DLMS)
**12a**	1000	4	0.70
**12b**	1000	4	0.70
**12c**	1000	4	0.71
**12d**	1000	4	0.65
**12e**	1000	4	0.85
**12f**	1000	4	0.82
**12g**	1000	4	0.83
**12h**	1000	4	0.76
**14a**	1000	4	−0.50
**14b**	1000	4	−0.43
**14c**	1000	4	−0.37
**14d**	1000	4	−0.44

**Table 6:** Topological polar surface area (TPSA) values of pyrazole–isatin **12a**–**h** and pyrazole–indole **14a**–**d**.

Pyrazole Conjugates	Topological Polar Surface Area (TPSA)
**12a**	104.18
**12b**	104.18
**12c**	113.41
**12d**	113.41
**12e**	104.18
**12f**	104.18
**12g**	113.41
**12h**	113.41
**14a**	97.96
**14b**	97.96
**14c**	107.20
**14d**	107.20

## Data Availability

Data are contained within this article or Supplementary File.

## Supplementary Materials

The following supporting information can be downloaded at: https://www.mdpi.com/article/10.3390/pharmaceutics17030293/s1, the procedures, the characterized spectral data, and charts for pyrazole–isatin conjugates **12a**–**h** and pyrazole–indole conjugates **14a**–**d**, the in vitro enzymatic assessment approaches, and statistical analysis details.
