# Supplementary material for: In Vitro Enzymatic and Computational Assessments of Pyrazole–Isatin and Pyrazole–Indole Conjugates as Anti-Diabetic, Anti-Arthritic, and Anti-Inflammatory Agents
Source: Pharmaceutics. 2025 Feb 23;17(3):293. doi: 10.3390/pharmaceutics17030293 (PMC11946580; doi:10.3390/pharmaceutics17030293)
Supplement: Supplementary file 1 [file pharmaceutics-17-00293-s001.zip › pharmaceutics-3484653-supplementary.pdf]

Supplementary File
For

In Vitro Enzymatic and Computational Assessments of Pyrazole-Isatin and Pyrazole-Indole Conjugates as Anti-Diabetic, Anti-Arthritic, and Anti-Inflammatory Agents

Ahmed M. Naglah [1,*], Abdulrahman A. Almehizia [1], Mohammed Ghazwani [2], Asma S. Al-Wasidi [3], Abdelrahman A. Naglah [4], Wael M. Aboulthana [5], and Ashraf S. Hassan [6,*]

[1] Drug Exploration & Development Chair (DEDC), Department of Pharmaceutical Chemistry, College of Pharmacy, King Saud University, Riyadh 11451, Saudi Arabia, mehizia@ksu.edu.sa
[2] Department of Pharmaceutics, College of Pharmacy, King Khalid University, P.O. Box 1882, Abha 61441, Saudi Arabia, myghazwani@kku.edu.sa
[3] Department of Chemistry, College of Science, Princess Nourah Bint Abdulrahman University, Riyadh 11671, Saudi Arabia, asalwasidi@pnu.edu.sa
[4] Faculty of Medicine, Zagazig University, Zagazig 44519, Egypt, boodynaglah@gmail.com
[5] Biochemistry Department, Biotechnology Research Institute, National Research Centre, Dokki, Cairo 12622, Egypt, wm.kamel@nrc.sci.eg
[6] Organometallic and Organometalloid Chemistry Department, National Research Centre, Dokki, Cairo 12622, Egypt
* Correspondence: anaglah@ksu.edu.sa (A.M.N.); as.el-salmoon@nrc.sci.eg (A.S.H.)

<u>**Pyrazole-isatin conjugates 12a-h**</u>

Procedure for synthesis of pyrazole-isatin conjugates 12a-h

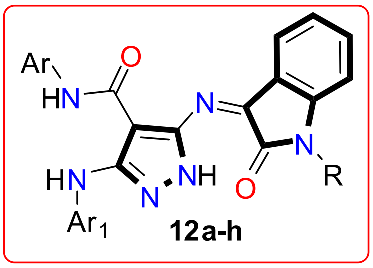

A mixture of 5-amino-pyrazoles **10a-d** (0.01 mol) with *N*-alkyl isatin **11a, b** (0.01 mol) {namely; 1-methylindoline-2,3-dione (**11a**) and 1-ethylindoline-2,3-dione (**11b**)} with a catalytic amount of glacial acetic acid (0.5 ml) in absolute ethanol (25 ml), the reaction mixture was refluxed for 1 hour and then left to cool. The solid product was filtered off, dried, and finally recrystallized from ethanol to afford target products **12a-h**.

5-(1-Methyl-2-oxoindolin-3-ylideneamino)-*N*-phenyl-3-(phenylamino)-1*H*-pyrazole-4-carboxamide (12a): M.p. 270-272 °C. ^{1}H NMR (DMSO-d_6, 400 MHz, δ ppm): 3.25 (s, 3H, NCH_3), 6.85 (t, 1H, J = 7.0 & 6.8 Hz, ArH), 7.04-7.15 (m, 4H, ArH), 7.34-7.38 (m, 4H, ArH), 7.58 (d, 2H, J = 7.7 & 7.7 Hz, ArH), 7.93 (d, 2H, J = 6.4 Hz, ArH), 8.99 (s, 1H, NH), 9.11 (d, 1H, J = 7.3 Hz, ArH), 11.16 (s, 1H, NH), 13.43 (s, 1H, NH). ^{13}C NMR (DMSO-d_6, 100 MHz, δ ppm): 26.30 (C, NCH_3), 95.28 (C, C_4-pyrazole), 109.75, 116.55, 118.31, 118.56, 122.41, 122.97, 128.89, 129.01, 129.50, 135.77, 138.13, 139.10, 147.94, 148.99 (18C, Ar), 158.86 (C, C_5-pyrazole), 159.01 (C, C_3-pyrazole), 162.14 (C, -N=C-), 162.29, 163.81 (2C, 2C=O). Anal. Calcd. (%) for $C_{25}H_{20}N_6O_2$ (436.47): C, 68.80; H, 4.62; N, 19.25. Found: C, 68.92; H, 4.55; N, 19.30 %.

5-(1-Methyl-2-oxoindolin-3-ylideneamino)-3-(phenylamino)-*N*-(4-methylphenyl)-1*H*-pyrazole-4-carboxamide (12b): M.p. > 300 °C. ^{1}H NMR (DMSO-d_6, 400 MHz, δ ppm): 2.27 (s, 3H, CH_3), 3.25 (s, 3H, NCH_3), 7.05 (t, 1H, J = 6.8 & 6 Hz, ArH), 7.10-7.16 (m, 4H, ArH), 7.30-7.38 (m, 4H, ArH), 7.59 (t, 1H, J = 7.6 & 6.5 Hz, ArH), 7.82 (d, 2H, J = 6.0 Hz, ArH), 9.06 (s, 1H, NH), 9.11 (d, 1H, J = 7.2 Hz, ArH), 11.07 (s, 1H, NH), 13.44 (s, 1H, NH). ^{13}C NMR (DMSO-d_6, 100 MHz, δ ppm): 20.47 (C, CH_3), 26.30 (C, NCH_3), 96.52 (C, C_4-pyrazole), 109.73, 118.29, 118.50, 119.53, 122.41, 129.27, 129.39, 129.51, 131.86, 135.75, 136.62, 139.53, 147.16 (18C, Ar), 148.99 (C, C_5-pyrazole), 153.11 (C, C_3-pyrazole), 153.51 (C, -N=C-), 161.96, 162.75 (2C, 2C=O). Anal. Calcd. (%) for $C_{26}H_{22}N_6O_2$ (450.49): C, 69.32; H, 4.92; N, 18.66. Found: C, 69.25; H, 5.00; N, 18.60 %.

3-(4-Methoxyphenylamino)-5-(1-methyl-2-oxoindolin-3-ylideneamino)-N-phenyl-1H-pyrazole-4-carboxamide (12c): M.p. 271-273 °C. ^{1}H NMR (DMSO-d_6, 400 MHz, δ ppm): 3.24 (s, 3H, NCH_3), 3.76 (s, 3H, OCH_3), 6.96 (d, 2H, J = 8.8 Hz, ArH), 7.05 (t, 1H, J = 7.3 & 8.8 Hz, ArH), 7.11 (t, 1H, J = 6.6 & 7.0 Hz, ArH), 7.13 (d, 1H, J = 7.1 Hz, ArH), 7.28 (d, 2H, J = 8.6 Hz, ArH), 7.35 (t, 2H, J = 8.2 & 7.9 Hz, ArH), 7.56 (t, 1H, J = 7.8 & 7.7 Hz, ArH), 7.94 (d, 2H, J = 7.8 Hz, ArH), 8.80 (s, 1H, NH), 9.10 (d, 1H, J = 7.5 Hz, ArH), 11.12 (s, 1H, NH), 13.15 (s, 1H, NH). ^{13}C NMR (DMSO-d_6, 100 MHz, δ ppm): 26.27 (C, NCH_3), 55.31 (C, OCH_3), 94.17 (C, C_4-pyrazole), 109.65, 114.74, 116.60, 118.44, 121.80, 122.34, 122.81, 128.87, 129.34, 132.05, 135.62, 139.27, 148.56, 148.91 (18C, Ar), 149.45 (C, C_5-pyrazole), 149.57 (C, C_3-pyrazole), 155.66 (C, -N=C-), 162.27, 163.88 (2C, 2C=O). Anal. Calcd. (%) for $C_{26}H_{22}N_6O_3$ (466.49): C, 66.94; H, 4.75; N, 18.02. Found: C, 67.00; H, 4.70; N, 18.00 %.

3-(4-Methoxyphenylamino)-5-(1-methyl-2-oxoindolin-3-ylideneamino)-*N*-(4-methylphenyl)-1*H*-pyrazole-4-carboxamide (12d): M.p. 262-263 °C. ^{1}H NMR (DMSO-d_6, 400 MHz, δ ppm): 2.27 (s, 3H, CH_3), 3.25 (s, 3H, NCH_3), 3.76 (s, 3H, OCH_3), 6.96 (d, 2H, J = 8.9 Hz, ArH), 7.09-7.16 (m, 4H, ArH), 7.28 (d, 2H, J = 8.2 Hz, ArH), 7.58 (t, 1H, J = 7.8 & 7.7 Hz, ArH), 7.82 (d, 2H, J = 7.5 Hz, ArH), 8.81 (s, 1H, NH), 9.10 (d, 1H, J = 6.7 Hz, ArH), 11.03 (s, 1H, NH), 13.15 (s, 1H, NH). ^{13}C NMR (DMSO-d_6, 100 MHz, δ ppm): 20.47 (C, CH_3), 26.29 (C, NCH_3), 55.32 (C, OCH_3), 94.20 (C, C_4-pyrazole), 109.68, 114.74, 116.60, 118.41, 119.45, 121.67, 122.36, 129.26, 131.70, 132.16, 135.63, 136.75, 148.50, 148.91 (18C, Ar), 149.39 (C, C_5-pyrazole), 149.53 (C, C_3-pyrazole), 155.48 (C, -N=C-), 162.10, 163.86 (2C, 2C=O). Anal. Calcd. (%) for $C_{27}H_{24}N_6O_3$ (480.52): C, 67.49; H, 5.03; N, 17.49. Found: C, 67.40; H, 5.10; N, 17.55 %.

5-(1-Ethyl-2-oxoindolin-3-ylideneamino)-*N*-phenyl-3-(phenylamino)-1*H*-pyrazole-4-carboxamide (12e): M.p. 274-276 °C. ^{1}H NMR (DMSO-d_6, 400 MHz, δ ppm): 1.24 (t, 3H, J = 7.2 & 7.1 Hz, $CH_2\underline{CH_3}$), 3.83 (q, 2H, J = 7.2 Hz, $\underline{CH_2}CH_3$), 6.86 (t, 1H, J = 7.6 & 6.6 Hz, ArH), 7.03-7.14 (m, 3H, ArH), 7.19 (d, 1H, J = 7.8 Hz, ArH), 7.24 (t, 1H, J = 8.3 & 7.3 Hz, ArH), 7.35-7.39 (m, 4H, ArH), 7.57 (t, 1H, J = 7.7 & 7.7 Hz, ArH), 7.93 (d, 2H, J = 7.7 Hz, ArH), 9.05 (s, 1H, NH), 9.10 (d, 1H, J = 7.2 Hz, ArH), 11.98 (s, 2H, 2NH). ^{13}C NMR (DMSO-d_6, 100 MHz, δ ppm): 14.10 (C, CH_3), 34.50 (C, NCH_2), 95.27 (C, C_4-pyrazole), 109.80, 116.72, 118.31, 119.60, 122.36, 123.02, 128.93, 129.54, 130.93, 135.83, 139.13, 141.54, 147.99, 148.44 (18C, Ar), 158.98 (C, C_5-pyrazole), 159.81 (C, C_3-pyrazole), 162.21 (C, -N=C-), 162.60, 163.41 (2C, 2C=O). Anal. Calcd. (%) for $C_{26}H_{22}N_6O_2$ (450.49): C, 69.32; H, 4.92; N, 18.66. Found: C, 69.25; H, 5.00; N, 18.60 %.

5-(1-Ethyl-2-oxoindolin-3-ylideneamino)-3-(phenylamino)-*N*-(4-methylphenyl)-1*H*-pyrazole-4-carboxamide (12f): M.p. 288-290 °C. ^{1}H NMR (DMSO-d_6, 400 MHz, δ ppm): 1.24 (t, 3H, J = 7.2 & 7.1 Hz, CH_2***CH_3***), 2.27 (s, 3H, CH_3), 3.83 (q, 2H, J = 7.2 Hz, ***CH_2***CH_3), 7.06-7.38 (m, 8H, ArH), 7.59 (t, 2H, J = 6.4 & 7.7 Hz, ArH), 7.83 (d, 2H, J = 7.6 Hz, ArH), 9.07 (s, 1H, NH), 9.15 (d, 1H, J = 7.2 Hz, ArH), 11.09 (s, 1H, NH), 13.45 (s, 1H, NH). ^{13}C NMR (DMSO-d_6, 100 MHz, δ ppm): 12.51 (C, CH_3), 20.47 (C, CH_3), 34.45 (C, NCH_2), 95.40 (C, C_4-pyrazole), 109.73, 118.48, 119.51, 122.49, 128.92, 129.27, 129.53, 131.83, 135.74, 136.65, 139.50, 141.10, 147.07, 147.95 (18C, Ar), 159.00 (C, C_5-pyrazole), 159.86 (C, C_3-pyrazole), 161.97 (C, -N=C-), 162.12, 163.46 (2C, 2C=O). Anal. Calcd. (%) for $C_{27}H_{24}N_6O_2$ (464.52): C, 69.81; H, 5.21; N, 18.09. Found: C, 69.75; H, 5.27; N, 18.00 %.

5-(1-Ethyl-2-oxoindolin-3-ylideneamino)-3-(4-methoxyphenylamino)-*N*-phenyl-1*H*-pyrazole-4-carboxamide (12g): M.p. 269-271 °C. ^{1}H NMR (DMSO-d_6, 400 MHz, δ ppm): 1.24 (t, 3H, J = 7.2 & 7.1 Hz, CH_2***CH_3***), 3.76 (s, 3H, OCH_3), 3.85 (q, 2H, J = 7.2 Hz, ***CH_2***CH_3), 6.96 (d, 2H, J = 8.8 Hz, ArH), 7.06 (t, 1H, J = 7.4 & 7.4 Hz, ArH), 7.12 (t, 1H, J = 7.7& 7.6 Hz, ArH), 7.21 (d, 1H, J = 7.8 Hz, ArH), 7.29 (d, 2H, J = 8.6 Hz, ArH), 7.36 (t, 2H, J = 8.0 & 7.7 Hz, ArH), 7.58 (t, 1H, J = 7.7 & 7.8 Hz, ArH), 7.95 (d, 2H, J = 7.9 Hz, ArH), 8.81 (s, 1H, NH), 9.15 (d, 1H, J = 7.6 Hz, ArH), 11.15 (s, 1H, NH), 13.18 (s, 1H, NH). ^{13}C NMR (DMSO-d_6, 100 MHz, δ ppm): 12.52 (C, CH_3), 34.46 (C, NCH_2), 55.32 (C, OCH_3), 94.19 (C, C_4-pyrazole), 109.75, 114.76, 116.75, 181.45, 121.88, 122.30, 122.84, 128.90, 129.63, 132.04, 135.73, 139.25, 147.92, 148.63 (18C, Ar), 149.48 (C, C_5-pyrazole), 149.63 (C, C_3-pyrazole), 155.69 (C, -N=C-), 162.28, 163.52 (2C, 2C=O). Anal. Calcd. (%) for $C_{27}H_{24}N_6O_3$ (480.52): C, 67.49; H, 5.03; N, 17.49. Found: C, 67.40; H, 5.10; N, 17.55 %.

5-(1-Ethyl-2-oxoindolin-3-ylideneamino)-3-(4-methoxyphenylamino)-*N*-(4-methylphenyl)-1*H*-pyrazole-4-carboxamide (12h): M.p. 240-241 °C. ^{1}H NMR (DMSO-d_6, 400 MHz, δ ppm): 1.24 (t, 3H, J = 7.2 & 7.1 Hz, CH_2***CH_3***), 2.27 (s, 3H, CH_3), 3.76 (s, 3H, OCH_3), 3.84 (q, 2H, J = 7.1 Hz, ***CH_2***CH_3), 6.95 (d, 2H, J = 8.8 Hz, ArH), 7.11 (t, 1H, J = 7.6 & 7.8 Hz, ArH), 7.15 (d, 2H, J = 8.3 Hz, ArH), 7.20 (d, 1H, J = 7.9 Hz, ArH), 7.27 (d, 2H, J = 8.6 Hz, ArH), 7.57 (t, 1H, J = 7.7 & 7.7 Hz, ArH), 7.83 (d, 2H, J = 8.0 Hz, ArH), 8.82 (s, 1H, NH), 9.14 (d, 1H, J = 7.6 Hz, ArH), 11.05 (s, 1H, NH), 13.17 (s, 1H, NH). ^{13}C NMR (DMSO-d_6, 100 MHz, δ ppm): 12.52 (C, CH_3), 20.47 (C, CH_3), 34.45 (C, NCH_2), 55.31 (C, OCH_3), 94.24 (C, C_4-pyrazole), 109.71, 114.75, 116.76, 181.41, 121.72, 122.27, 129.27, 129.62, 131.68, 132.10, 135.67, 136.76, 147.89, 148.50 (18C, Ar), 149.46 (C, C_5-pyrazole), 149.57 (C, C_3-pyrazole), 155.63 (C, -N=C-), 162.12, 163.50 (2C, 2C=O). Anal. Calcd. (%) for $C_{28}H_{26}N_6O_3$ (494.54): C, 68.00; H, 5.30; N, 16.99. Found: C, 68.10; H, 5.25; N, 16.90 %.

Pyrazole-indole conjugates 14a-d

Procedure for synthesis of pyrazole-indole conjugates 14a-d

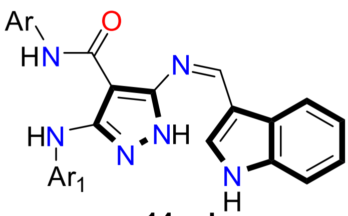

14a-d

A mixture of 5-amino-pyrazoles **10a-d** (0.01 mol) with 1*H*-indole-3-carbaldehyde (**13**) (0.01 mol, 1.45 g) and a catalytic amount of glacial acetic acid (0.5 ml) in absolute ethanol (25 ml), the reaction mixture was refluxed for 1 hour and then left to cool. The solid product was filtered off, dried, and finally recrystallized from ethanol to afford compounds **14a-d**.

5-((1H-Indol-3-yl)methyleneamino)-N-phenyl-3-(phenylamino)-1*H*-pyrazole-4-carboxamide (14a): M.p. 266-268 °C. H NMR (DMSO-d_6, 400 MHz, δ ppm): 6.87 (t, 1H, J = 7.3 & 7.3 Hz, ArH), 7.07 (t, 1H, J = 7.4 & 7.4 Hz, ArH), 7.24-7.37 (m, 6H, ArH), 7.57-7.65 (m, 5H, ArH), 8.33 (s, 1H, indole), 8.35 (d, 1H, J = 7.9 Hz, ArH), 9.00 (s, 1H, -N=CH-), 9.06 (s, 1H, NH), 10.03 (s, 1H, NH), 12.30 (s, 2H, 2NH). ^{13}C NMR (DMSO-d_6, 100 MHz, δ ppm): 92.05 (C, C_4-pyrazole), 112.99, 114.34, 116.44, 119.42, 119.82, 121.23, 122.07, 123.33, 123.90, 124.35, 128.63, 128.98, 129.02, 137.73, 138.57, 141.36 (20C, Ar), 149.74 (C, C_5-pyrazole), 152.03 (C, C_3-pyrazole), 158.89 (C, -N=C-), 163.29 (C, C=O). Anal. Calcd. (%) for $C_{25}H_{20}N_6O$ (420.47): C, 71.41; H, 4.79; N, 19.99. Found: C, 71.35; H, 4.85; N, 20.05 %.

5-((1*H*-Indol-3-yl)methyleneamino)-3-(phenylamino)-*N*-(4-methylphenyl)-1*H*-pyrazole-4-carboxamide (14b): M.p. 276-278 °C. ^{1}H NMR (DMSO-d_6, 400 MHz, δ ppm): 2.27 (s, 3H, CH_3), 6.87 (t, 1H, J = 7.3 & 7.3 Hz, ArH), 7.14 (d, 2H, J = 8.3 Hz, ArH), 7.24-7.31 (m, 3H, ArH), 7.35 (t, 1H, J = 7.1 & 7.0 Hz, ArH), 7.53 (d, 2H, J = 8.4 Hz, ArH), 7.57 (d, 2H, J = 8.2 Hz, ArH), 7.60 (d, 1H, J = 8.1 Hz, ArH), 8.33 (s, 1H, indole), 8.34 (d, 1H, J = 7.6 Hz, ArH), 9.00 (s, 1H, -N=CH-), 9.05 (s, 1H, NH), 9.96 (s, 1H, NH), 12.29 (s, 2H, 2NH). ^{13}C NMR (DMSO-d_6, 100 MHz, δ ppm): 20.38 (C, CH_3), 92.02 (C, C_4-pyrazole), 112.94, 114.28, 116.34, 119.35, 121.18, 122.02, 123.84, 124.27, 128.98, 129.31, 132.27, 135.99, 137.66, 139.39, 141.34, 149.44 (20C, Ar), 153.15 (C, C_5-pyrazole), 155.63 (C, C_3-pyrazole), 158.66 (C, -N=C-), 163.09 (C, C=O). Anal. Calcd. (%) for $C_{26}H_{22}N_6O$ (434.49): C, 71.87; H, 5.10; N, 19.34. Found: C, 71.95; H, 5.00; N, 19.40 %.

5-((1*H*-Indol-3-yl)methyleneamino)-3-(4-methoxyphenylamino)-*N*-phenyl-1*H*-pyrazole-4-carboxamide (14c): M.p. 272-274 °C. ^{1}H NMR (DMSO-d_6, 400 MHz, δ ppm): 3.73 (s, 3H, OCH_3), 6.90 (d, 2H, J = 8.2 Hz, ArH), 7.07 (t, 1H, J = 7.4 & 7.4 Hz, ArH), 7.26 (t, 1H, J = 7.2 & 7.5 Hz, ArH), 7.30-7.36 (m, 4H, ArH), 7.60 (d, 1H, J = 8.1 Hz, ArH), 7.64 (d, 2H, J = 7.6 Hz, ArH), 8.33 (s, 1H, indole), 8.35 (d, 2H, J = 7.9 Hz, ArH), 8.77 (s, 1H, -N=CH-), 9.03 (s, 1H, NH), 9.98 (s, 1H, NH), 12.30 (s, H, NH), 12.60 (s, H, NH). ^{13}C NMR (DMSO-d_6, 100 MHz, δ ppm): 55.21 (C, OCH_3), 91.67 (C, C_4-pyrazole), 112.92, 114.29, 117.42, 119.30, 121.18, 122.03, 123.19, 123.82, 124.31, 128.93, 137.65, 138.62, 152.83, 155.31 (20C, Ar), 156.20 (C, C_5-pyrazole), 156.60 (C, C_3-pyrazole), 158.92 (C, -N=C-), 163.27 (C, C=O). Anal. Calcd. (%) for $C_{26}H_{22}N_6O_2$ (450.49): C, 69.32; H, 4.92; N, 18.66. Found: C, 69.25; H, 5.00; N, 18.60 %.

5-((1*H*-Indol-3-yl)methyleneamino)-3-(4-methoxyphenylamino)-*N*-(4-methylphenyl)-1*H*-pyrazole-4-carboxamide (14d): M.p. 258-260 °C. ^{1}H NMR (DMSO-d_6, 400 MHz, δ ppm): 2.26 (s, 3H, CH_3), 3.72 (s, 3H, OCH_3), 6.89 (d, 2H, J = 9.0 Hz, ArH), 7.13 (d, 2H, J = 8.3 Hz, ArH), 7.25 (t, 1H, J = 7.8 & 7.9 Hz, ArH), 7.34 (t, 1H, J = 8.2 & 8.1 Hz, ArH), 7.48 (d, 2H, J = 8.5 Hz, ArH), 7.53 (d, 2H, J = 8.4 Hz, ArH), 7.59 (d, 1H, J = 8.1 Hz, ArH), 8.31 (s, 1H, indole), 8.34 (d, 1H, J = 7.7 Hz, ArH), 8.78 (s, 1H, -N=CH-), 9.05 (s, 1H, NH), 9.96 (s, 1H, NH), 12.26 (s, 2H, 2NH). ^{13}C NMR (DMSO-d_6, 100 MHz, δ ppm): 20.37 (C, CH_3), 55.22 (C, OCH_3), 91.54 (C, C_4-pyrazole), 112.90, 114.28, 144.33, 118.23, 119.28, 121.18, 121.94, 123.78, 124.28, 129.30, 132.16, 136.09, 137.52, 137.64, 150.02, 153.20 (20C, Ar), 153.24 (C, C_5-pyrazole), 153.31 (C, C_3-pyrazole), 158.37 (C, -N=C-), 163.12 (C, C=O). Anal. Calcd. (%) for $C_{27}H_{24}N_6O_2$ (464.52): C, 69.81; H, 5.21; N, 18.09. Found: C, 69.75; H, 5.25; N, 18.00 %.

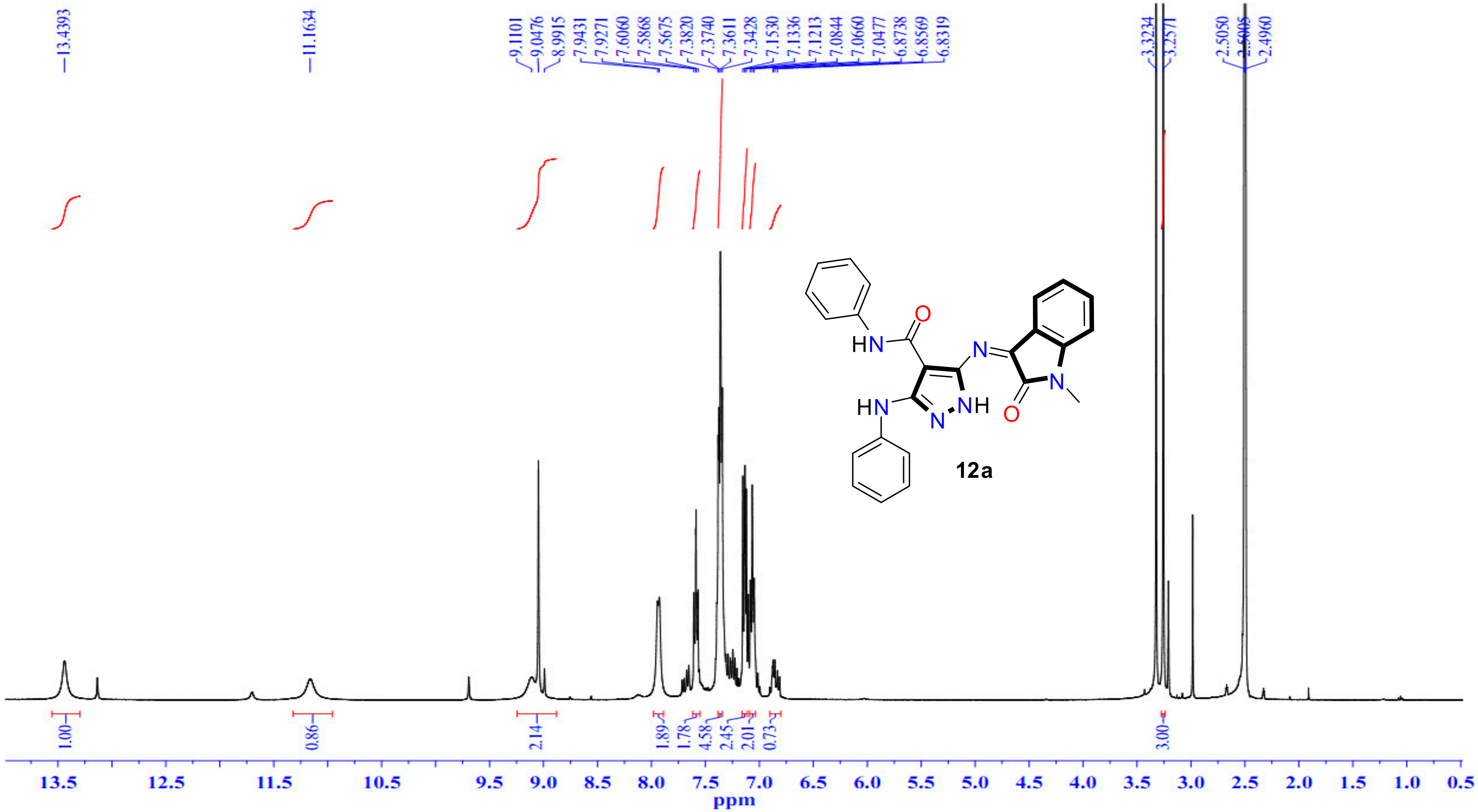

The ^{1}H NMR (400 MHz) spectrum of compound 12a

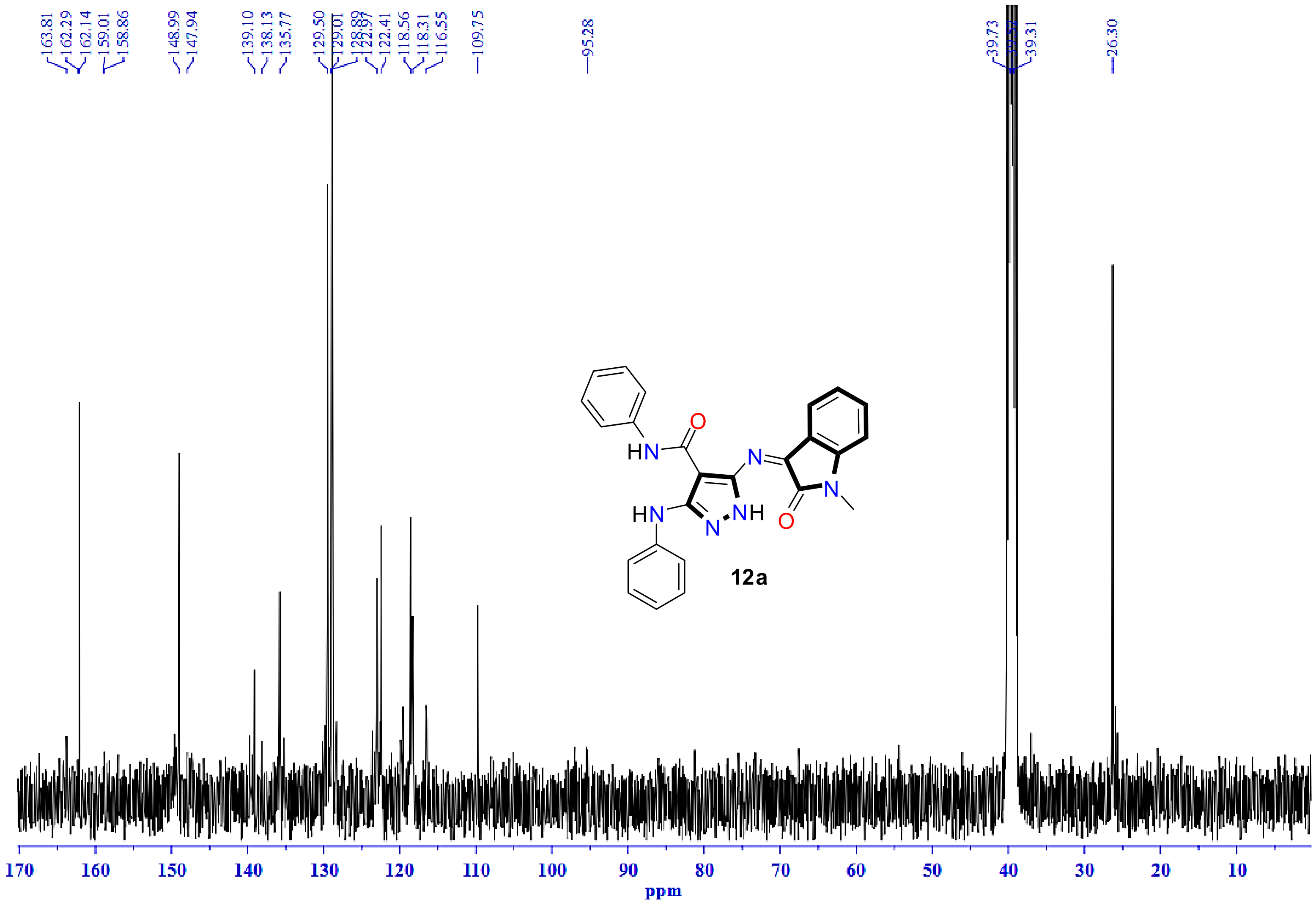

The ^{13}C NMR (100 MHz) spectrum of compound 12a

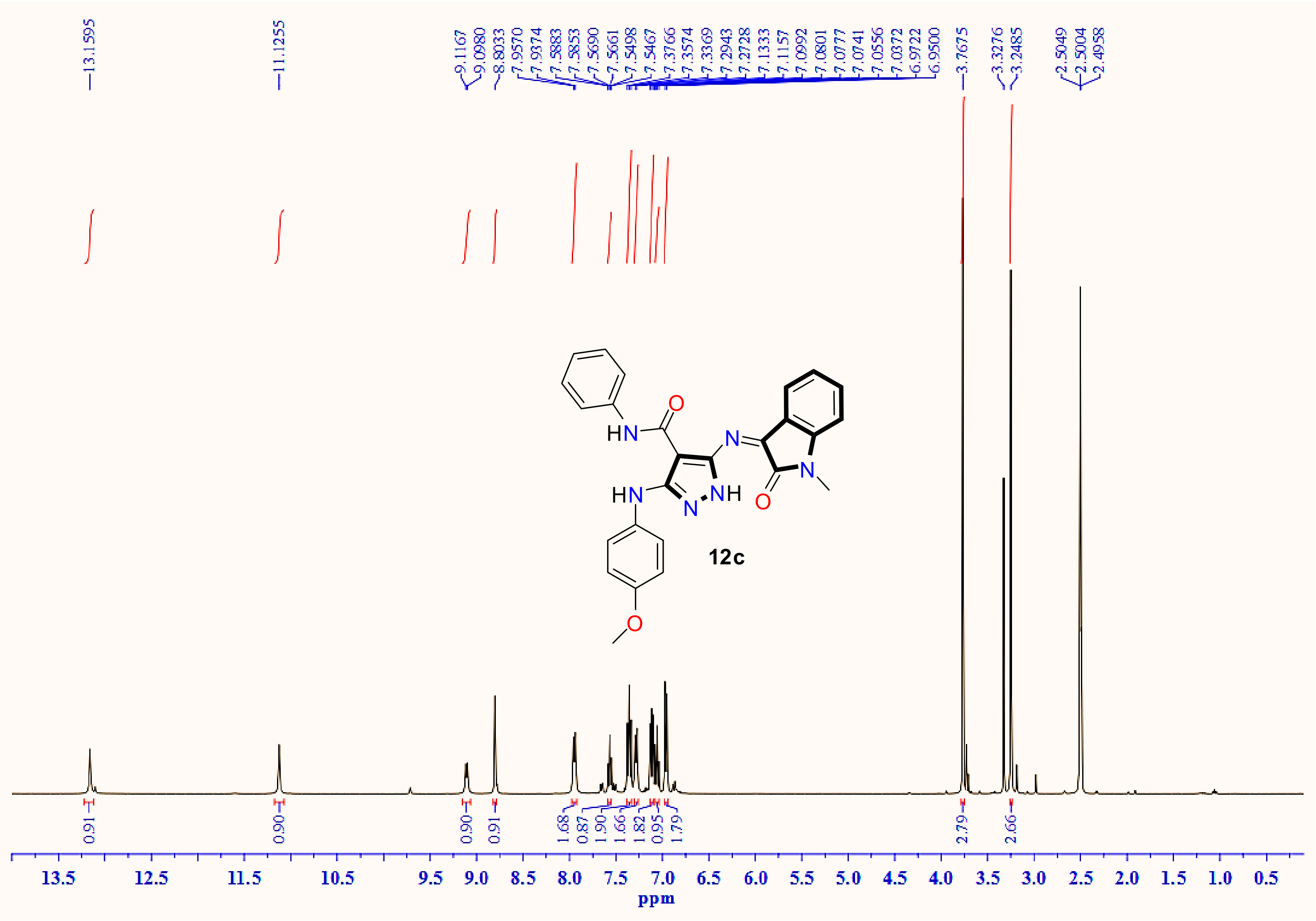

The ^{1}H NMR (400 MHz) spectrum of compound 12c

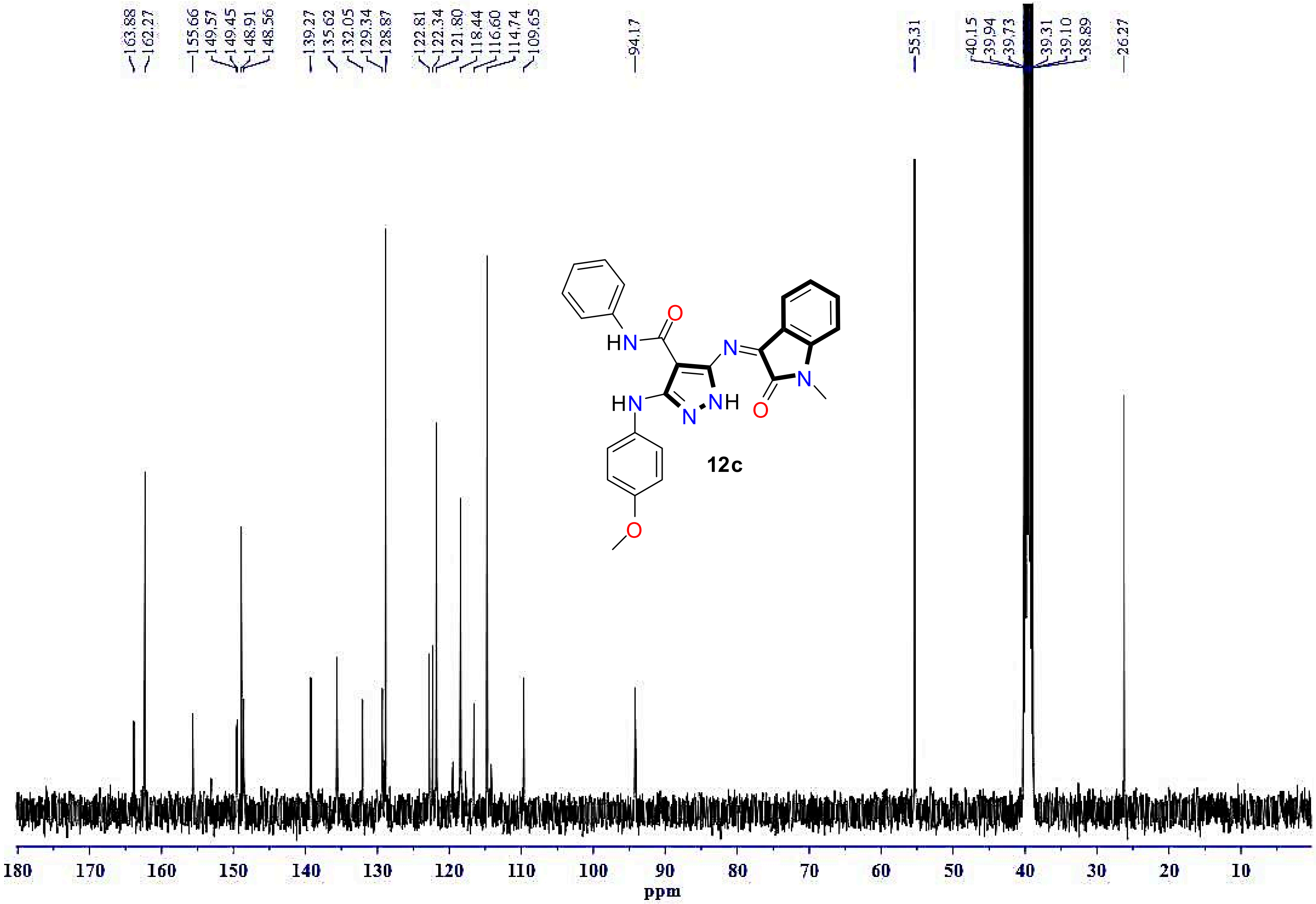

The ^{13}C NMR (100 MHz) spectrum of compound 12c

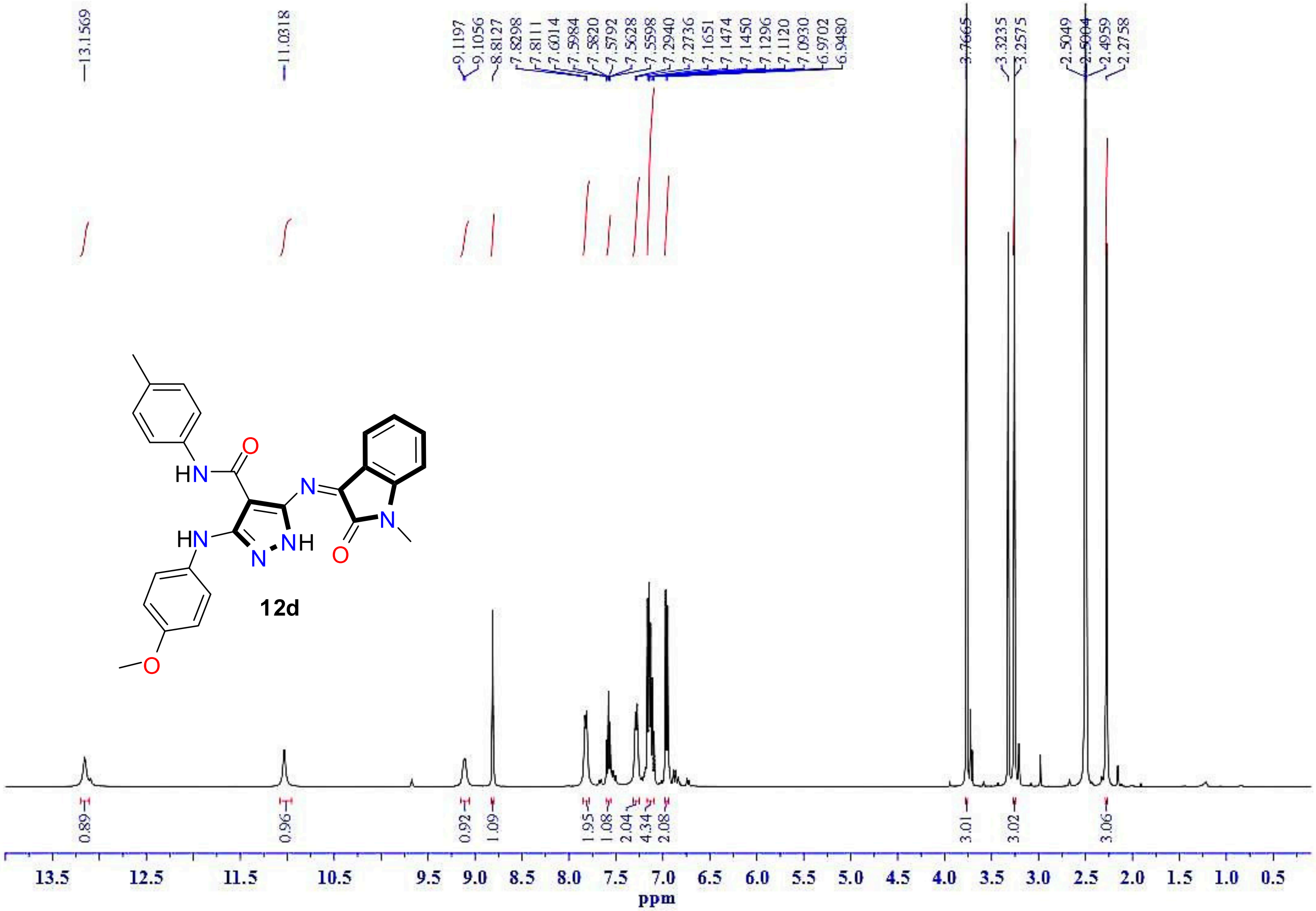

The ^{1}H NMR (400 MHz) spectrum of compound 12d

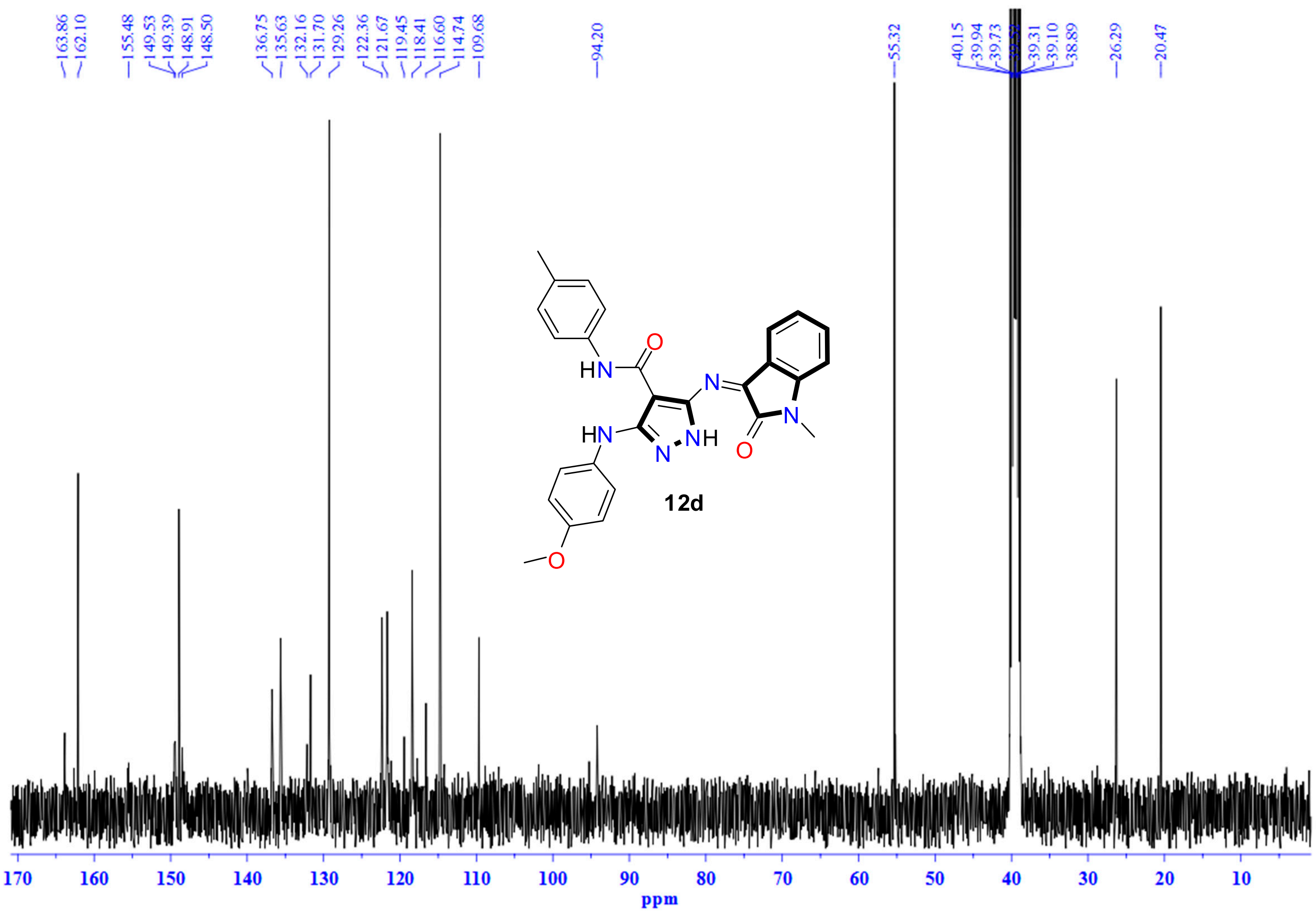

The ^{13}C NMR (100 MHz) spectrum of compound 12d

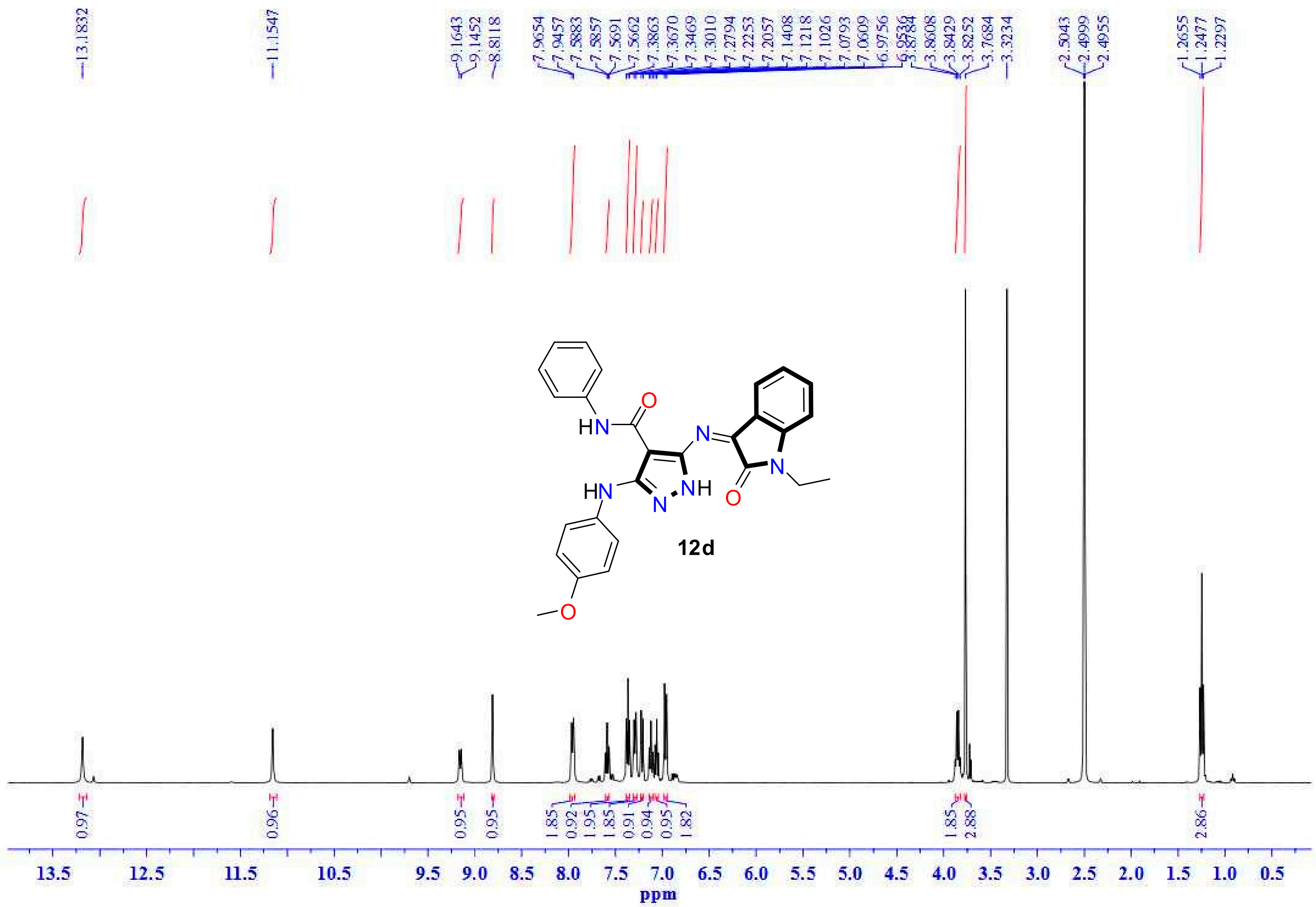

The ^{1}H NMR (400 MHz) spectrum of compound 12g

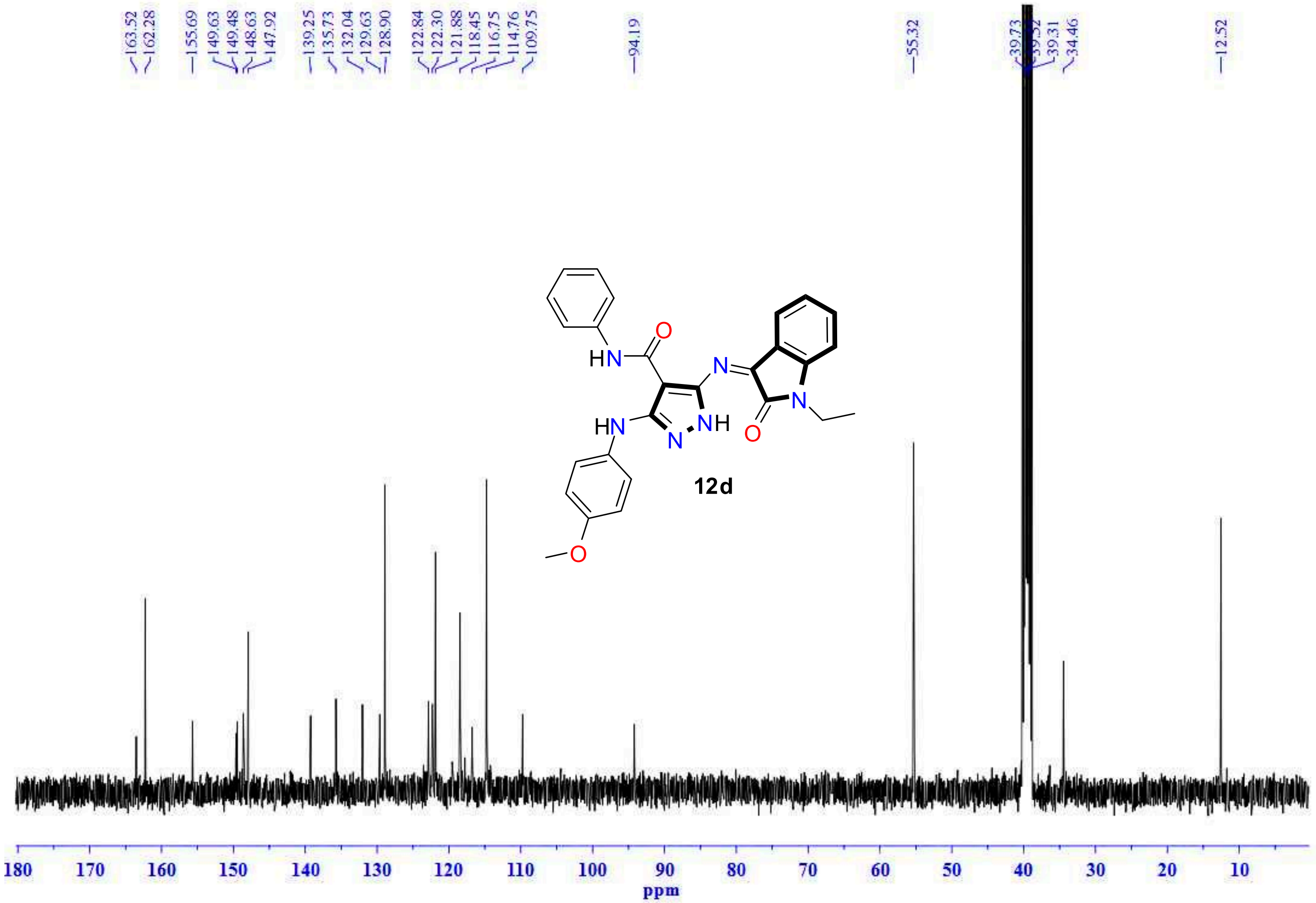

The ^{13}C NMR (100 MHz) spectrum of compound 12g

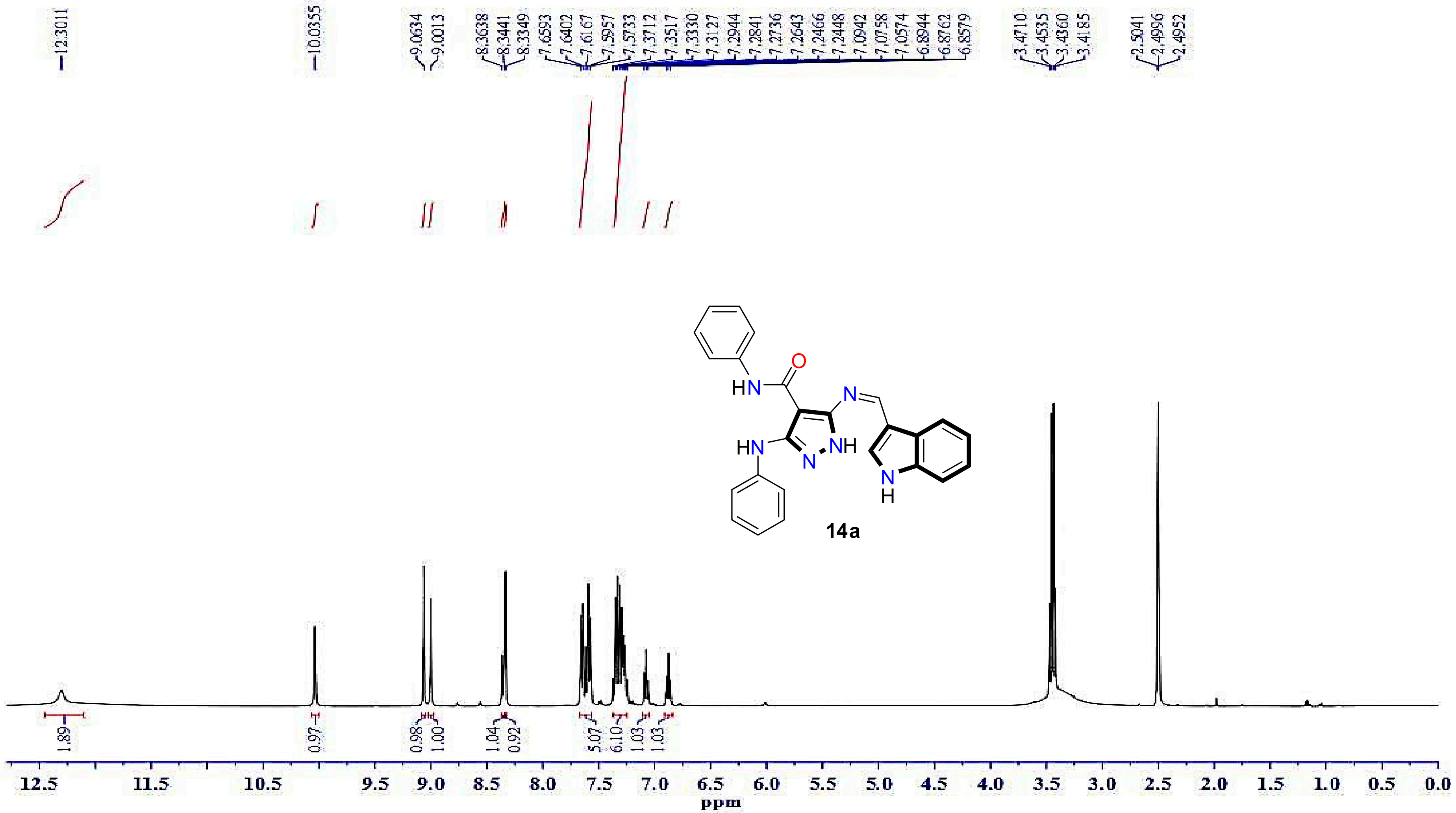

The ^{1}H NMR (400 MHz) spectrum of compound 14a

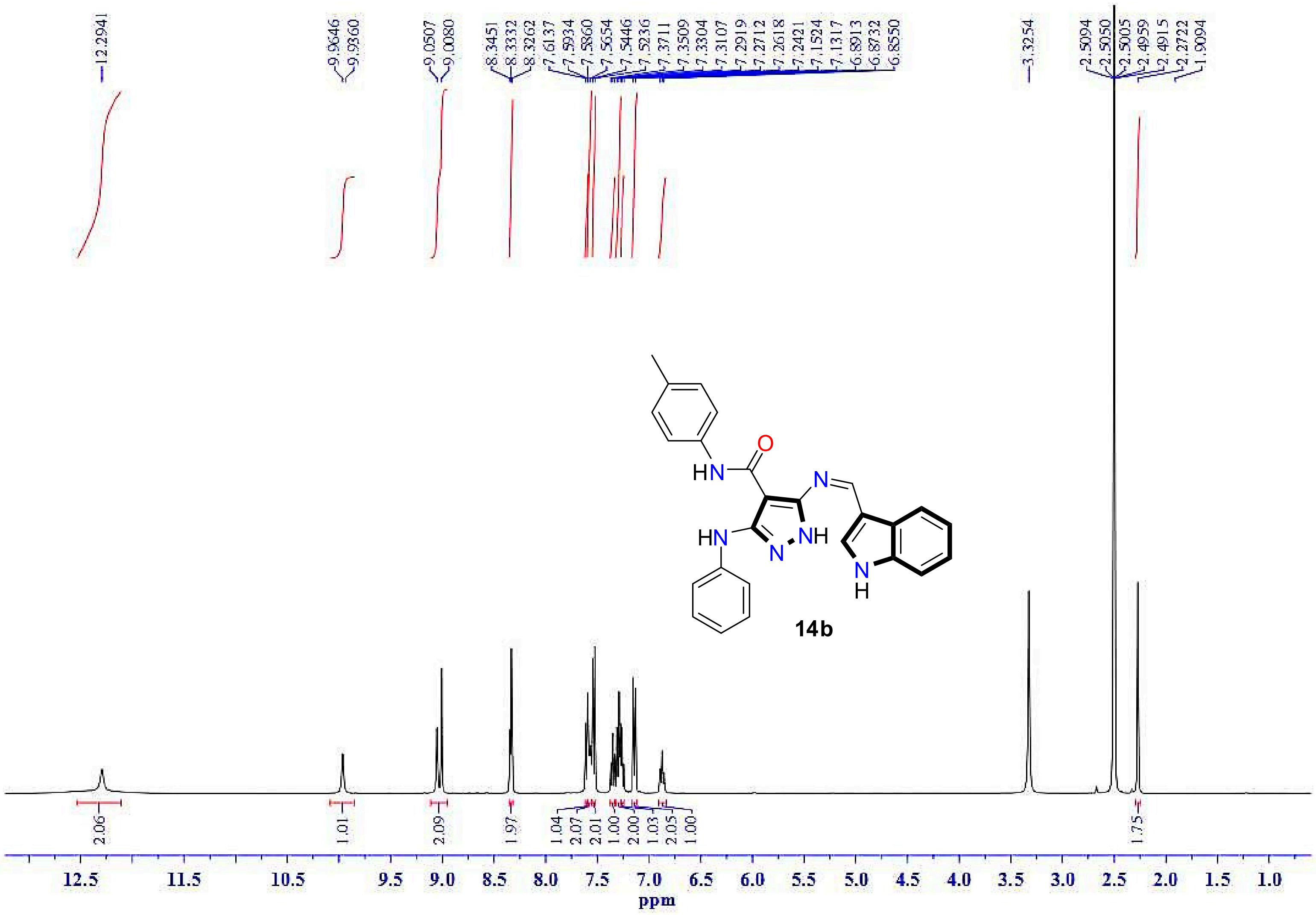

The ^{1}H NMR (400 MHz) spectrum of compound 14b

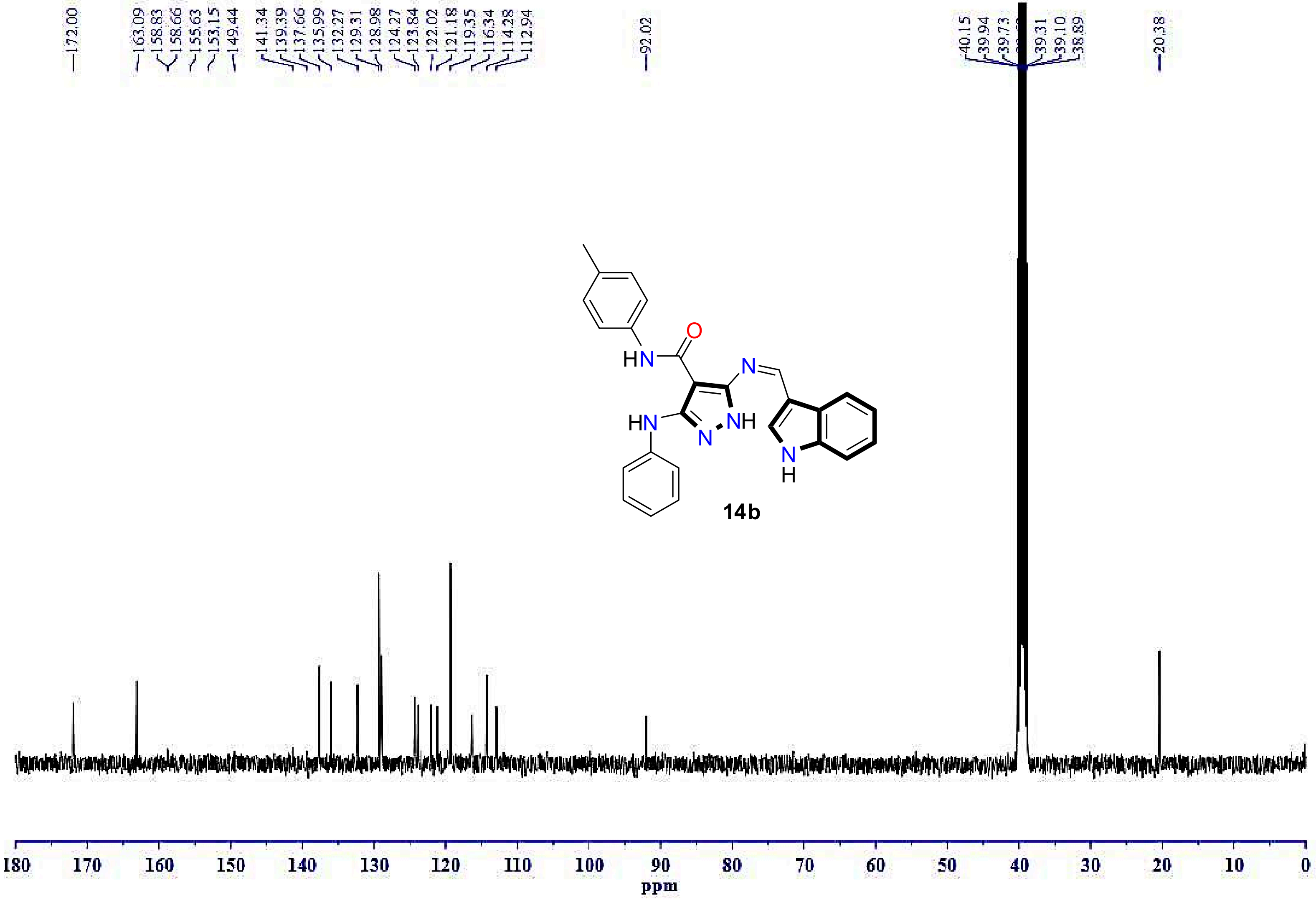

The ^{13}C NMR (100 MHz) spectrum of compound 14b

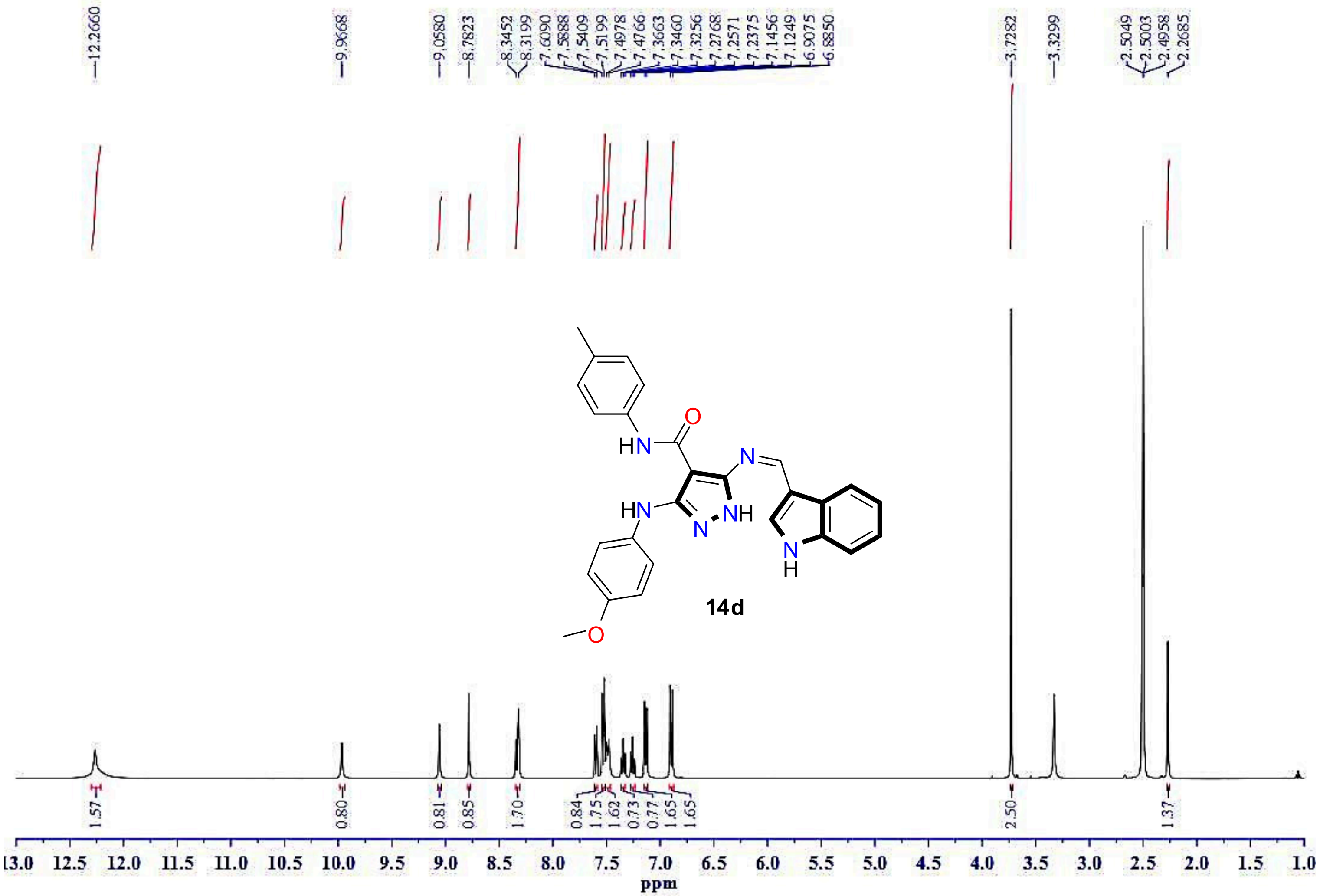

The ¹H NMR (400 MHz) spectrum of compound 14d

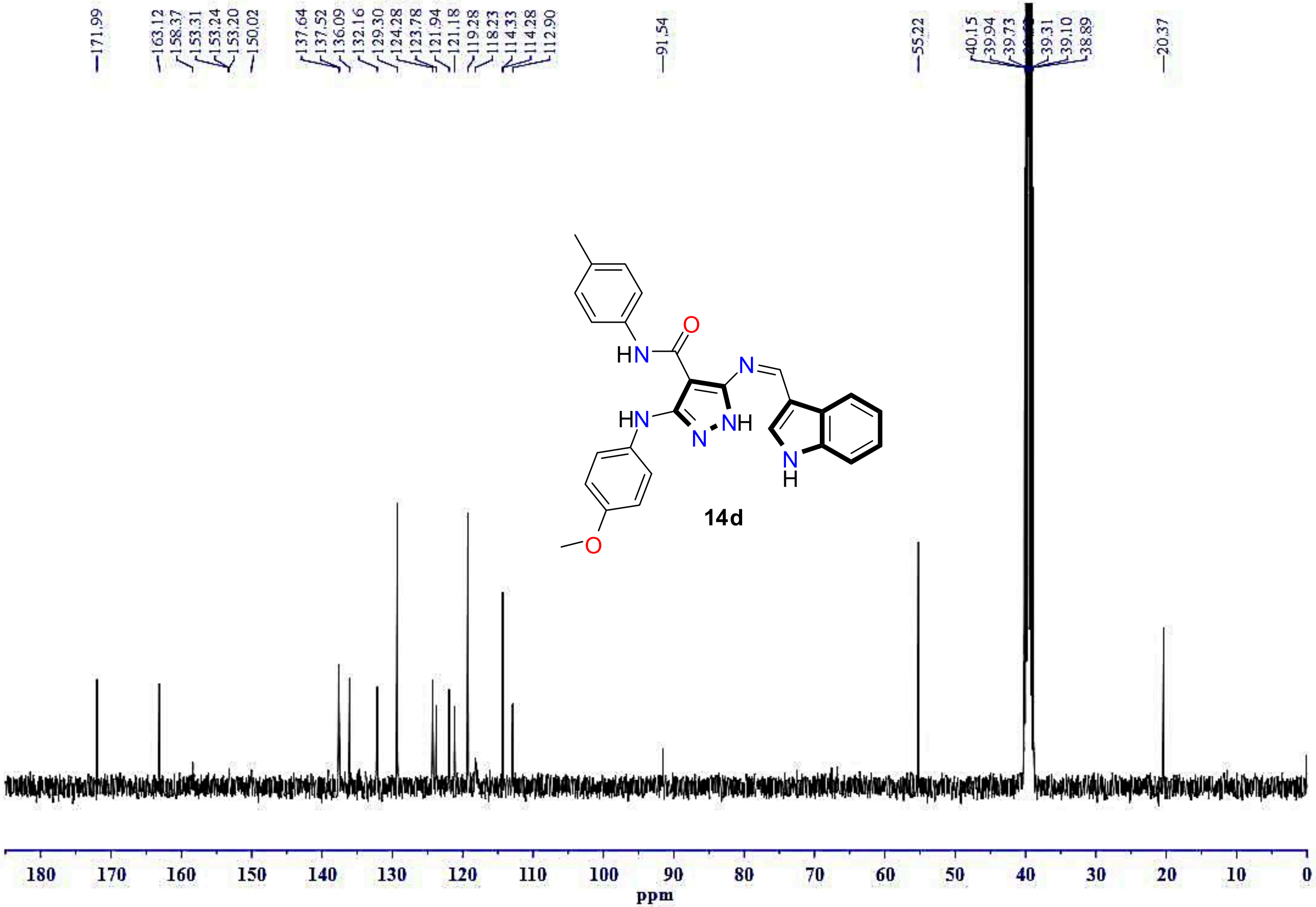

The ^{13}C NMR (100 MHz) spectrum of compound 14d

In vitro enzymatic assessments

Anti-diabetic activity

This assay involved calculating the inhibition percentage (%) of α-amylase enzyme using method based on the technique demonstrated by Wickramaratne *et al.* with Acarbose as the standard drug. During the assay, 0.5 ml of the test solution was combined with 0.5 ml of α-amylase solution (0.5 mg/ml) and buffer (Na_2HPO_4/NaH_2PO_4 (0.02 M), NaCl (0.006 M) at pH 6.9) to create concentrations ranging from 25 to 800 μg/mL. The mixture was then left at room temperature for 10 minutes before adding 200 μL of starch solution (1% in water (w/v) buffer (Na_2HPO_4/NaH_2PO_4 (0.02 M), NaCl (0.006 M) at pH 6.9)). The reaction was stopped by adding 200 μL of DNSA (coloring) reagent (12 g of sodium potassium tartrate tetrahydrate in 8.0 mL of 2 M NaOH and 20 mL of 96 mM of DNSA solution). The test tubes were then placed in a boiling water bath (100 °C) for 10 minutes and the mixture was cooled to room temperature and diluted with 5 mL of distilled water. The absorbance was measured at 540 nm using a UV-Visible spectrophotometer. The IC_{50} of each tested sample was calculated by plotting a curve using a series of sample concentrations against the percent of α-amylase inhibition.

The inhibition percentage (%) of the α-glucosidase enzyme was determined using the method proposed by Pistia-Brueggeman and Hollingsworth with Acarbose as the standard drug. Five μL of the α-glucosidase solution (10 units mL^{-1}, $0.1moll^{-1}$ potassium phosphate buffer, pH 6.8) was pre-mixed with 10 μL of the sample solution at different concentrations (in 10% DMSO) in 620 μL of 0.1 $moll^{-1}$ potassium phosphate buffer (pH 6.8). After incubation at 37.5 °C for 20 minutes, 10 μL of p-nitro phenyl glucopyranoside (pNPG, 10 $mmoll^{-1}$) as a substrate was added to the mixture to start the reaction. The reaction mixture was then incubated at 37.5°C for 30 minutes, followed by the addition of 650 μL of 1 $moll^{-1}$ Na_2CO_3 solution to terminate the reaction. The amount of released product (p-nitro phenol) was measured at 410 nm using a UV spectrometer (UV-2550, Shimadzu, Japan) to estimate the enzymatic activity. The inhibition assay was performed in triplicate for all tests. The IC_{50} of each tested sample was calculated by plotting a curve using a series of sample concentrations against the percent of α-glucosidase inhibition.

Anti-arthritic activity

In the anti-arthritic activity study, this assay involved determining the percentage of protein denaturation and proteinase inhibition using diclofenac sodium as the standard non-steroidal anti-inflammatory drug, as prepared according to **Meera *et al.*** The protein denaturation percentage was measured by mixing 0.5mL of the test control solution, prepared

by combining 0.45 mL of bovine serum albumin (BSA) (5% w/v aqueous solution) with 0.05 mL of distilled water. Then, 0.05 mL of the test solution was added to 0.45 mL of distilled water to form the product control (0.5 mL). The different samples (test solution) and diclofenac sodium (standard) were used. The pH value in all prepared solutions was adjusted to 6.3 using HCl (1N). All the samples were incubated at 37 °C for 20 min, and the temperature was then increased to 57 °C, maintaining the samples at that degree for 3 min. After cooling, 2.5 mL of phosphate buffer was added to the prepared solutions. The absorbance was determined at 416 nm using a UV-Visible spectrophotometer. The percentage of protein denaturation inhibition can be calculated. Proteinase inhibitory activity was assessed by combining the test sample (1 mL) with a reaction mixture containing 0.06 mg trypsin dissolved in 1 mL of 20 mM Tris HCl buffer (pH 7.4). The mixture was then incubated for 5 minutes at 37°C, followed by the addition of 1 mL of casein (0.8% w/v). After an additional 20 minutes of incubation, 2 mL of perchloric acid (70%) was added to stop the reaction. The cloudy suspension was then centrifuged, and the absorbance of the supernatant was measured at 210 nm against buffer as the blank. The percentage of proteinase inhibitory activity was then calculated.

Anti‑inflammatory activity

In vitro anti-inflammatory evaluation was performed through inhibition of two isoenzymes cyclooxygenase COX-1 and COX-2 (ovine/human), along with 5-LOX enzyme (human recombinant).

COX‑1 and COX‑2 inhibition assay has been performed by means of COX-1 and COX-2 kit (Cayman, No.: 560131), where different known concentrations of the tested compounds were added separately to a mixture of 10 µL of COX-1 or COX-2 and 0.1 M HCl buffer, left for incubation at room temperature for 10 min. After that, 10µL of arachidonic acid, fifty µL HCl and Ellman's reagent have been added. The absorbance has been determined at UV-410 nm alongside blank; IC_{50} has been determined via linear regression.

5‑Lipoxygenase inhibition assay was carried out by 5-LOX kit (No. 437996, Sigma-Aldrich), where different concentrations of the tested compounds were added to 90 µL from 5-LOX, 100 µL of de chromogen, then 10 µL from arachidonic acid was added and shaken for 10 min, the absorbance has been determined at UV-490 nm compared to blank. IC50 has been calculated through linear regression.

Statistical analysis

All values were expressed in tables and figures as mean ± SE calculated from three replicates (n=3). The Statistical Package for the Social Sciences (SPSS for Windows®, Version 11.0, 2001, SPSS Inc., Chicago, USA) was used to conduct a one-way analysis of variance (ANOVA) to assess both positive and negative correlations among *in vitro* biological activities. A significance level of a "*p*" value <0.05 was used to determine significant correlations.
